# Trends and gaps in the use of citizen science derived data as input for species distribution models: A quantitative review

**DOI:** 10.1371/journal.pone.0234587

**Published:** 2021-03-11

**Authors:** Mariano J. Feldman, Louis Imbeau, Philippe Marchand, Marc J. Mazerolle, Marcel Darveau, Nicole J. Fenton

**Affiliations:** 1 Centre d’étude de la forêt, Institut de Recherche sur les Forêts (IRF), Université du Québec en Abitibi-Témiscamingue (UQAT), Rouyn-Noranda, Québec, Canada; 2 Département des sciences du bois et de la forêt, Centre d’étude de la forêt, Faculté de foresterie, de géographie et de géomatique, Université Laval, Québec City, Québec City, Canada; 3 Ducks Unlimited Canada, Québec City, Québec City, Canada; Instituto Federal de Educacao Ciencia e Tecnologia Goiano - Campus Urutai, BRAZIL

## Abstract

Citizen science (CS) currently refers to the participation of non-scientist volunteers in any discipline of conventional scientific research. Over the last two decades, nature-based CS has flourished due to innovative technology, novel devices, and widespread digital platforms used to collect and classify species occurrence data. For scientists, CS offers a low-cost approach of collecting species occurrence information at large spatial scales that otherwise would be prohibitively expensive. We examined the trends and gaps linked to the use of CS as a source of data for species distribution models (SDMs), in order to propose guidelines and highlight solutions. We conducted a quantitative literature review of 207 peer-reviewed articles to measure how the representation of different taxa, regions, and data types have changed in SDM publications since the 2010s. Our review shows that the number of papers using CS for SDMs has increased at approximately double the rate of the overall number of SDM papers. However, disparities in taxonomic and geographic coverage remain in studies using CS. Western Europe and North America were the regions with the most coverage (73%). Papers on birds (49%) and mammals (19.3%) outnumbered other taxa. Among invertebrates, flying insects including Lepidoptera, Odonata and Hymenoptera received the most attention. Discrepancies between research interest and availability of data were as especially important for amphibians, reptiles and fishes. Compared to studies on animal taxa, papers on plants using CS data remain rare. Although the aims and scope of papers are diverse, species conservation remained the central theme of SDM using CS data. We present examples of the use of CS and highlight recommendations to motivate further research, such as combining multiple data sources and promoting local and traditional knowledge. We hope our findings will strengthen citizen-researchers partnerships to better inform SDMs, especially for less-studied taxa and regions. Researchers stand to benefit from the large quantity of data available from CS sources to improve global predictions of species distributions.

## Introduction

Species distribution models have become a widely used tool in ecology and have tackled diverse scientific issues at different spatial and temporal scales in recent years [1–3]. Understanding the association between the occurrence of species and environmental conditions is a first step in addressing questions about species distributions, abundances and habitat preferences [4, 5]. In fact, knowledge on species distributions is paramount in order to develop biodiversity conservation and management strategies [6]. Current global-scale issues such as climate and land-use changes have also increased the need to be able to predict the distribution of migratory or invasive species across a landscape. The fundamental theory behind species distribution models (SDMs, hereafter) assumes that the presence of a species in a given location depends on the environment, which implies that ecologists are able to estimate past, current, or future species distributions based on the environmental characteristics of unsurveyed locations [3, 5, 7, 8]. Specifically, SDMs link information about the presence of a species to the environmental variables of their known locations, and apply statistical models to predict the spatial distribution of species [4, 5, 9]. Consequently, three major components can be identified in any framework for SDMs: species presence data, landscape or environmental data, and a statistical model that links the first two components [4, 5, 10]. Species distribution models are widely used in both fundamental science and applied sciences in biogeography, evolution, dispersal, migration, species invasion, meta-population, conservation, and climate change [3]. For example, SDMs have shown their value to assess species invasions [11], to predict spatial changes in response to climate change or land-use changes [12–14], or to assess the suitability of possible conservation areas [15–17].

Modelling the distribution of species usually requires a large amount of information collected over multiple years of standardized fieldwork [18–20]. However, the long-term collection of broad-scale information on a wide range of species is prohibitively expensive [21]. Yet, for some taxa, an impressive volume of data collected using mostly non-standardized protocols is currently available on online portals, through efforts collectively labelled as citizen science (CS, hereafter). Globally, a huge variety of CS programs are currently being implemented involving a wide range of taxa [22]. Nevertheless, CS data are still challenging to analyze due to the intrinsic issues of non-standardized protocols that can affect the credibility and quality of the data [23, 24].

Issues within CS datasets arise from the large number of observations that vary in quality when used as a source for research [25, 26]. Previous studies have tackled the different sources of error and bias in CS data [27–29]. Firstly, CS datasets are typically biased towards human population centers, areas that are easy to access, protected areas, or regions frequented by active observers [30–32]. These problems lead to disparities in effort between over-sampled and under-sampled areas [19, 29, 30, 33–35]. Secondly, geographical coverage of CS data can be biased towards well-financed and more industrialized countries, mainly in North America and Europe [25, 32, 36]. These two regions contribute substantially more data than any other region in the Global Biodiversity Information Facility (GBIF) database [37–41]. Consequently, a large proportion of samples occur in a restricted geographical extent, controlled by administrative borders. This results in a non-representative sample of species’ distribution. Thirdly, over time, the observation and reporting protocols can change. For example, the Audubon Christmas Bird Count at its start in 1900 aimed at offering an alternative to hunting on Christmas Day morning, with a loose survey protocol. The date for conducting the count became flexible over the years. For example, it was conducted during a window of 7 days in 1940 [42]. The sampling window then expanded to 12 days in 1966 [43] and the protocol was further standardized to collect a snapshot of wintering birds around Christmas time [44]. The survey period expanded again in 2000, this time to 23 days [45]. Unfortunately, changes in survey protocols were often poorly documented [46, 47]. Fourthly, CS observations can be taxonomically biased because volunteers are usually attracted to large and common species, to species that are brightly colored and easy to detect, and to more charismatic groups [28, 40, 47, 48]. This taxonomical disparity results in more information on relatively well-known groups than for under-reported groups. Finally, another source of variation in CS programs includes the variation in skill and expertise among observers, primarily due to the participation of a wide range of volunteers [49, 50]. The quality of observations depends on the ability of observers to correctly detect and identify species. This inter-observer sampling variation increases for species that are harder to identify [49, 51, 52]. Bias and precision associated with each of these five sources of variation can influence predictions of future trends. A major challenge is to account for these issues in species distribution models [53, 54].

Despite these issues regarding data quality, the use of CS has increased in recent years in different fields of study [55, 56]. For instance, CS is used in astronomy to classify galaxy images or to search for signals in radio data, and in atmospheric sciences to record the quality of air, soil, and water [55, 57, 58]. However, the main application of CS is in conservation and ecology to monitor species occurrence [39, 59]. Past reviews have focused on how CS contributes to biodiversity monitoring [39], global change [60], and conservation biology [61]. However, considering the increasing prevalence of CS in ecological studies, it is critical to gain a better understanding of how this data source contributes to peer-reviewed research and to what extent CS can fill gaps in under-represented species and locations. Furthermore, the degree to which scientists already use CS data to build SDMs is not well documented. Understanding the contributions of different forms of CS that provide data for SDMs should help to better allocate research efforts in the future and outline specific strategies to increase the usefulness of CS.

The main objective of this review was to quantify the variation and gaps in the use of CS as an input for modelling species distribution. To achieve this objective, we assessed the current strengths in the use of CS in SDMs and identified partiality and under-use relative to taxa, regions, and data acquisition methods. We formulated three research questions: (1) What is the trend in the use of CS data for SDMs over the last decade? We expected the number of papers using CS data to have increased at a faster rate than the SDM field as a whole given the increasing contribution of citizen science in different field studies; (2) Is there variation across regions, taxa, and types of data used? We anticipated that because volunteers behave differently according to the region and group of interest, the set of papers would reflect clear preferences towards regions that are easy to access and groups that are visually appealing to volunteers. However, it is expected that these preferences will gradually fade due to the growing diversification of initiatives and platforms worldwide over the last decade; (3) What are the information gaps and how can research needs be met in the near future? We expected that new approaches of collecting data for sensitive species and under-sampled locations play an important role for filling research gaps.

## Materials and methods

### Paper selection

We used the Scopus search engine to conduct a literature review of peer-reviewed papers focusing on species distribution models that used citizen science. Our search spanned a period of 10 years, considering papers from 2010, when the “citizen science” term was widely accepted by several authors [58, 62, 63], until 17 October 2019. We searched for papers using the following combination of keywords: (“citizen science” OR “public participation” OR “community monitoring program” OR “participatory monitoring”) AND (“species distribution model” OR “predictive model” OR “distribution map” OR “invasive species” OR “occupancy model” OR “occurrence” OR “migration” OR “climate change”). The integration of citizen science data and advances in occupancy modelling allows researchers to build species distribution models that account for imperfect detection probability. Indeed, several authors encourage their use for a variety of species [59, 64, 65]. For our review, papers using occupancy models were included only when they were used for mapping species distribution.

### Data collection

**Fig 1:**
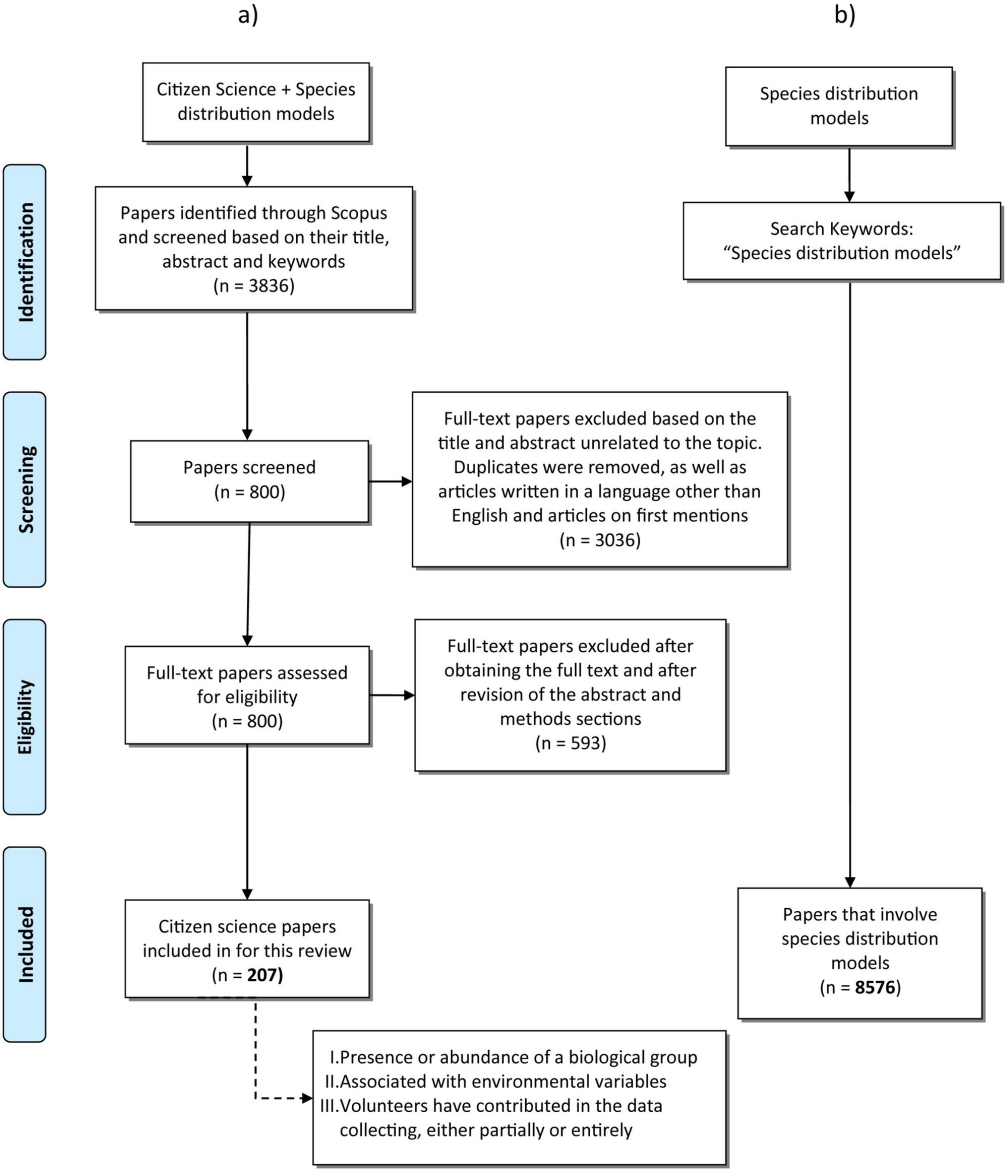
Flow chart of paper selection for a) the citizen science (CS) papers and b) for the entire species distributions models (SDMs) field. All 207 papers in a) are listed in S1 Table. *From*: Moher D, Liberati A, Tetzlaff J, Altman DG, The PRISMA Group (2009). *P*referred *R*eporting *I*terns for *S*ystematic Reviews and *M*eta-*A*nalyses: The PRISMA Statement. PLoS Med 6(7): e1000097. doi:10.1371/journal.pmed1000097 **For more information, visit** www.prisma-statement.org.

From the first Scopus search, a total of 3,836 papers were screened based on their title, abstract and keywords (Fig 1a). Those not related with either citizen science (CS) or species distribution models (SDMs) were dismissed. The remaining 800 papers were reduced after a further revision of abstracts and methodologies. We excluded papers written in languages other than English (n = 4), all review papers, and also papers using data gathered by volunteers but without applications of SDMs (e.g., first report of a species or new occurrence data). To consider a given paper as relevant for our review, each of the following three conditions had to be met: the data included the presence or abundance of a biological group, the data were collected by volunteers (either partially or entirely), and a statistical method was applied to assess relationships with environmental data (Fig 1a). Our search resulted in 207 papers from peer-reviewed journals. These papers formed the basis of the analyses presented herein. Some papers that used CS as a data source for SDM are not in the Scopus database and were certainly missed. Nevertheless, we consider that this database is a representative sample that covers most of the peer-reviewed journals of world science at present within the SDM field in the last decade. Details and extracted information about all papers included in our review are listed in (S1 Table). This list may not reflect the full influence of CS programs in SDMs, but only their contribution to published articles.

From each paper, the following information was extracted: (1) year of publication; (2) focal taxa; (3) source of data or platform used (if any); (4) country and region where the data was taken; (5) scope or central objective of the study—when appearing in the title, abstract, or keywords, including species conservation, land-use changes, biogeography, habitat suitability, population trends climate change, invasive species or migration; (6) data type used (presence-only, presence-absence, or abundance); (7) statistical approach used, and 8) the method of collecting CS data (opportunistic data, count data, community-based monitoring, historical records, local ecological knowledge, or trained volunteers). In order to assess the contribution of CS to SDMs over the last decade, we compiled papers within Scopus by using the keywords “species distribution models” to obtain the number of papers in this field (Fig 1b). This search engine seeks matches within the title, abstract, keywords, and indexed keywords and returned 8576 papers.

### Data analyses

#### Contribution of citizen science to species distribution models

We tested for differences in the rate of increase of CS-SDM papers and the overall number of SDM papers using generalized linear models (GLM) with a Poisson distribution that included an interaction term between the year and type of paper (CS-SDM vs SDMs). We expected papers using CS data to have increased at a faster rate than the SDM field as a whole.

#### Taxonomic groups

In order to assess the representation of CS within the biological groups, each paper was categorized within the following taxonomic groups: invertebrates, plants and fungi (including bryophytes and lichens), fish, reptiles, amphibians, mammals, and birds. We used a chi-square test to first compare the number of CS papers with data on each group observed to the numbers expected based on the proportion of species in each group according to the Catalogue of Life [66] (accession date April 2020). A second chi-square test was used to compare the number of occurrences for each taxa contained in the GBIF dataset (accession date 23 November 2020). To compare the observed (CS) and expected proportions for each of the five taxonomic groups, we constructed a logistic regression model excluding the intercept to estimate the logit of the probability that a taxa *t* appears in a CS study:

$$\text{logit}(p_{t})=\beta_{t}$$ (1)

We then calculated a Z-score from the difference between *β*_*t*_ and the logit of *E*_*t*_, the expected proportion for that taxa, scaled by the standard error of *β*_*t*_:

$$Z_{t}=\frac{\beta_{t}-logit(E_{t})}{SE\beta_{t}}$$ (2)

We obtained a two-tailed p-value for the null hypothesis that the observed proportion was equal to the expected proportion by comparing *Z*_*t*_ to the standard normal distribution. We excluded papers that focused on more than one taxonomic group to meet assumptions of statistical independence of observations. For invertebrates, only the taxonomic orders represented in our CS set of papers were analyzed (Lepidoptera, Odonata, Hymenoptera, Coleoptera and Mollusca). The remaining invertebrate groups were not represented or were in very low numbers in our review (e.g., spiders and hemiptera).

#### Geographic regions

Papers were individually classified into country and continent of origin of the CS data, including Africa, Asia, Eastern Europe, Western Europe, Oceania, North America, Central America, and South America. To assess if these regions were over or under-sampled in the CS papers set, we used a one-sample chi-square test to compare the number of CS papers in each region to the number expected based on the proportion of the Earth’s land area covered by each region (from http://www.worlddata.info; accessed 29.11.19). Then, we compared these observed and expected proportions for each of these geographic regions. Using the same strategy as above, we constructed a logistic regression model that excluded the intercept to estimate the logit of the probability that the region_*y*_ appears in a CS study:

$$\text{logit}(p_{y})=\beta_{y}$$ (3)

We then calculated a Z-score from the difference between *β*_*y*_ and the logit of *E*_*y*_, the expected proportion for that region, scaled by the standard error of *β*_*y*_:

$$Zy=\frac{\beta_{y}-logitE_{y}}{SE\beta_{y}}$$ (4)

We compared the *Z*_*y*_ against the standard normal distribution. Papers that focused on more than one region were excluded to meet assumptions of statistical independence of the observations. All statistical analyses were performed in R version 3.5.0 [67].

## Results and discussion

### Year of publication

**Fig 2:**
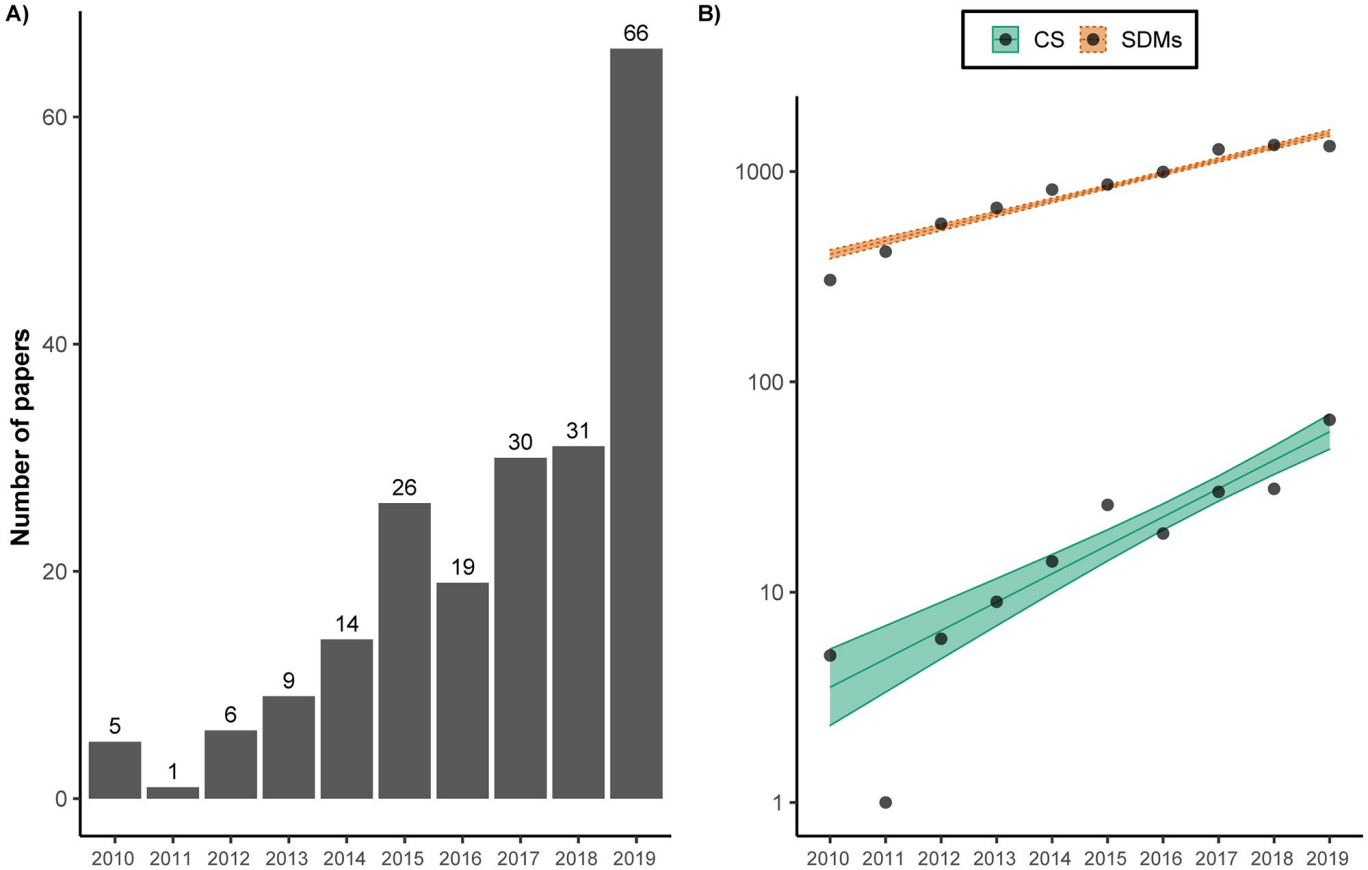
(a) Annual number of papers that have used species distribution models (SDMs) with citizen science (CS) data; (b) generalized linear model with Poisson distribution of the total papers using SDMs (blue) and the papers using CS data (red) across the 10-year period covered by our review (difference in slopes: -0.16, Z = -5.4, P < 0.001), resulting in publication growth of 16% for SDMs and 36% for CS on average per year.

As we expected, our analysis indicates that the use of CS data in the peer-reviewed SDM literature has increased in frequency over the past 10 years (Fig 2a). Numerous authors have indicated the increase in publications using different types of CS data [55, 56, 60, 68, 69], but also the growing rate of SDMs in publications [70, 71]. Our analysis shows that the use of CS in SDMs has grown approximately twice as fast as the number of papers using SDMs in general (publication increases of 16% for SDMs and 36% for CS on average per year). In addition, given its peak in 2019 with 66 papers (Fig 2a), the next few years may extend the rapid growth of the use of CS in SDMs.

### Taxonomic groups

**Fig 3:**
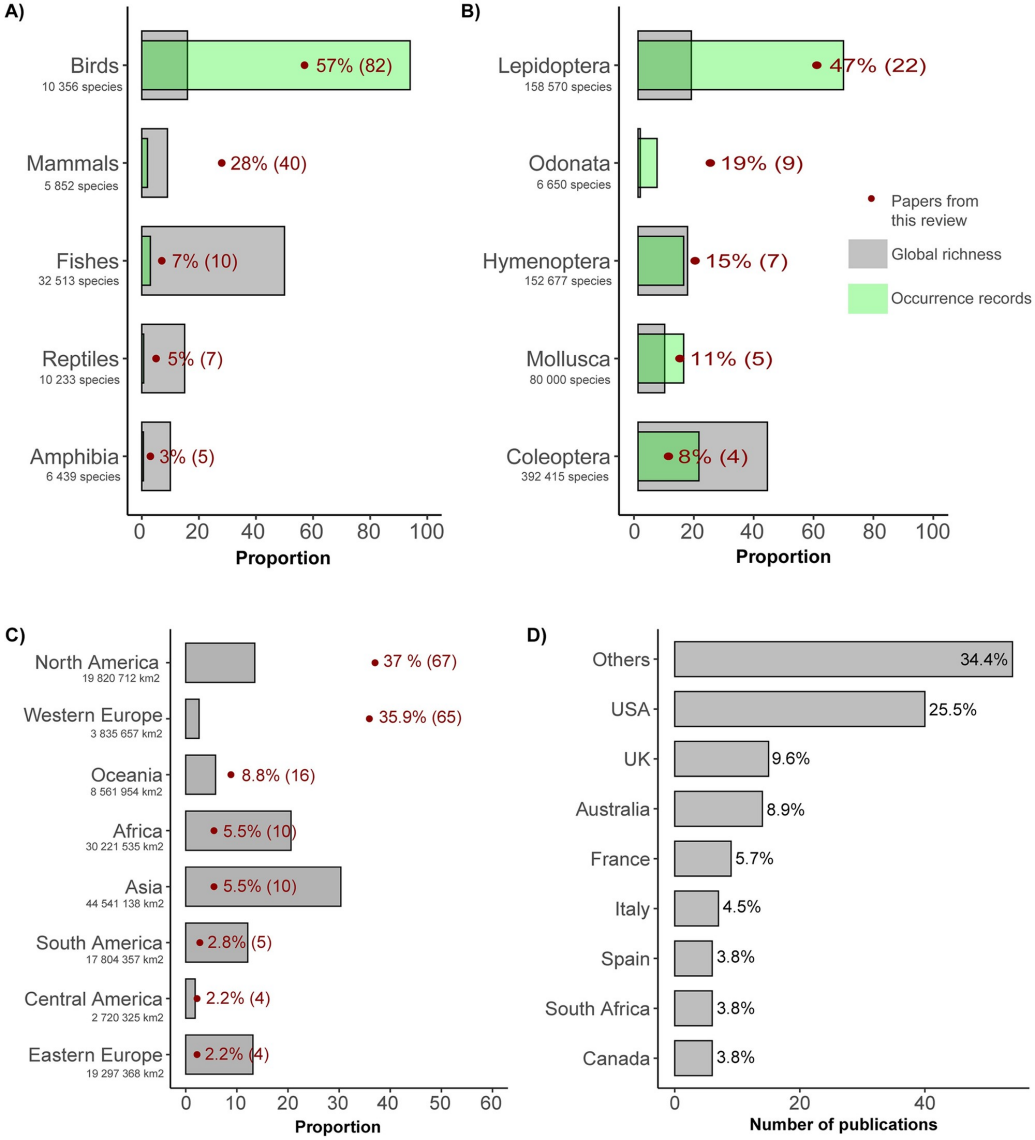
Proportion of citizen science (CS) papers from this review (red point) relative to the proportion of global richness in the Catalogue of Life (grey bars) by taxa and number of global occurrence in GBIF (green bars), for (a) vertebrates and (b) invertebrates; (c) proportion of CS papers by data collection region (red dotted lines) relative to each region’s fraction of the Earth’s land area (grey bars); and (d) proportion of CS papers by country.

Following our predictions for the more visually appealing species, there were marked variations among taxonomic groups, with birds (n = 83; 41%), invertebrates (n = 47; 23,3%), and mammals (n = 39; 19.3%) being the main taxa studied. Reptiles (n = 12; 5.9%), fish (n = 11; 5.4%) and amphibians (n = 10; 4.9%) received less attention (Fig 3a). This taxonomic preference towards bird species was previously noted by other authors [39, 55, 60], and is reflected in occurrence records from GBIF (94%; Fig 3a).

Compared to their global richness, vertebrate groups were unequally represented in the CS papers (χ^2^ = 262.77, df = 4, p < 0.001). Specifically, birds (*Z*_*birds*_ = 11.6; p < 0.001) and mammals (*Z*_*mammals*_ = 6.8; p < 0.001) were over-represented, whereas amphibians (*Z*_*amphibians*_ = -2.3; p = 0.02), reptiles (*Z*_*reptiles*_ = -8.1; p < 0.001) and fish (*Z*_*fish*_ = -7.7; p < 0.001) were under-represented in CS papers compared to their estimated global richness in the Catalogue of Life database (Fig 3). These differences were mostly driven by the interest of the volunteers collecting the data, which results in over-representation of charismatic taxa relative to groups generating less interest [72]. However, compared to the global occurrence records (number of occurrences per group instead of richness), mammals (*Z*_*mammals*_ = 15.9; p < 0.001), reptiles (*Z*_*reptiles*_ = 11.34; p < 0.001), amphibians (*Z*_*amphibians*_ = 4.1; p < 0.001) and fish (*Z*_*fish*_ = 2.7; p = 0.006) were over-represented in CS papers. In contrast, birds were largely under-represented compared to their occurrence records (*Z*_*birds*_ = -13.9; p < 0.001) since nearly all occurrence records were concentrated within this group (94% in Fig 3a). Such results suggest a great imbalance between research interests and availability of CS data, especially for amphibians, reptiles and fishes, which are over-represented in publications as compared to their occurrence data but under-represented compared to their estimated global species richness. Thus, there is a need to increase data acquisition on a wider range of species by volunteers on these specific groups containing several lesser-known species, with poorly defined distributions and conservation status. In the case of birds, reducing structured sampling efforts in areas already well-covered by volunteers may help redirect limited monitoring resources to sample less populated areas or to target more cryptic species. Filling such information gaps may also require local and traditional knowledge or community-based monitoring approaches, especially if research knowledge gaps also meet local concerns.

We found differences between the observed proportion of invertebrates in our CS data set and the proportion expected based on global richness (χ^2^ = 93.08, df = 4, p < 0.001). Lepidoptera (*Z*_*lepidoptera*_ = 4.4; p < 0.001) and Odonata (*Z*_*odonata*_ = 5.9; p < 0.001) were over-sampled relative to their global richness (Fig 3b). Only Coleoptera (*Z*_*coleoptera*_ = -4.1; p < 0.001) were under-sampled. The proportion of papers on Mollusca (*Z*_*mollusca*_ = 0.49; p = 0.62) and Hymenoptera (*Z*_*hymenoptera*_ = -0.29; p = 0.77) did not differ from the proportion expected from global species richness. The remaining invertebrate orders in the Catalogue of Life database did not occur or were in very low numbers in the set of CS papers studied. Considering occurrences records, proportions between our number of CS papers and the GBIF global dataset did not differ: Lepidoptera (*Z*_*lepidoptera*_ = -0.5; p = 0.58), Mollusca (*Z*_*mollusca*_ = 0.08; p = 0.93), Coleoptera (*Z*_*coleoptera*_ = -1.03; p = 0.3), Odonata (*Z*_*odonata*_ = 1.9; p = 0.051) and Hymenoptera (*Z*_*hymenoptera*_ = 1.01 p = 0.3). Such results suggest a better balance between research interests and availability of CS data for invertebrates than for vertebrates.

The plant and fungi group included papers involving vascular plants (n = 14), fungi (n = 3), lichens (n = 1), and bryophytes (n = 1; S1 Table). Considering the known number of species in each group according to the Catalogue of Life database (vascular plants: 348,000 species; fungus: 140,000 species; bryophytes 16,000 species), plant taxonomic groups were remarkably under-represented in CS papers (n = 13; 8% of all groups in this review). The major obstacle could be that identifying plants up to species level in the field is sometimes complex, even for expert botanists [73, 74]. Plant identification is time consuming for several families, requires significant botanical skills, and can be frustrating for non-expert volunteers [74]. In addition, there is not as strong a tradition for botanists in sharing observations using online portals, compared to animal databases. Nonetheless, plant initiatives seem to be highly attractive to the general public. Millions of observations are produced and stored in broad databases such as GBIF, iNaturalist, and in particular botanic platforms such as Pl@ntNet [75], Project Bud Burst [76], or Plant Watch Canada [77]. Several authors recommend using this information collected from volunteers for the early detection and control of invasive species [78, 79], or to improve the performance of models [79–81]. Nevertheless, our review confirms a notable under-use of plant, fungi, lichen and bryophyte public databases in the last decade in papers that model the distribution of species, as compared to the interest that they generate in CS programs.

### Geographic coverage

Our review identified strong geographic biases in CS sampling efforts (χ^2^ = 1374.5, df = 7, p < 0.001). While Western Europe (*Z*_*WesternEurope*_ = 20.5; p < 0.001) and North America (*Z*_*NorthAmerica*_ = 7.7; p < 0.001) were over-sampled relative to their fraction of the planet’s land area, Africa (*Z*_*Africa*_ = -4.4; p < 0.001), Asia (*Z*_*Asia*_ = -6.1; p < 0.001), South America (*Z*_*SouthAmerica*_ = -3.6; p < 0.001) and Eastern Europe (*Z*_*EasternEurope*_ = -3.8; p < 0.001) were under-sampled (Fig 3c). Oceania (*Z*_*Oceania*_ = 1.6; p = 0.11) and Central America (*Z*_*CentralAmerica*_ = 0.3; p = 0.77) were sampled proportionally to their area. At the country level, most of the papers using CS data were from USA (n = 40; 25.5%), the UK (n = 15; 9.5%), Australia (n = 14; 8.9%), France (n = 9; 5.7%), Italy (n = 7; 4.5%), South Africa, Spain and Canada (n = 6; 3.8%; Fig 3d).

Such a strong geographic inclination toward Europe and North America has already been indicated by several authors [39, 41, 82]. Others also revealed the same pattern of CS being predominantly conducted in Europe and North America, but with a greater number of studies in South and Central America [16, 32] than reported in our study. This geographical disparity of CS-based papers among regions is likely influenced by three factors. First, North America and Europe host more developed countries, which have more funding available for research [48], and consequently tend to publish more. Some of these countries have traditional national platforms such as the National Biodiversity Gateway (NBN) in the United Kingdom (containing around 127 million records), the Atlas of Living Australia (ALA; containing 87,179,824 records on 19^th^ April 2020), or the Sweden Species Gateway (containing around 60,000 species). Individual country platforms share characteristics associated with successful CS programs that contributed more to global biodiversity monitoring. These platforms receive important support by national governments and are linked to well-funded institutions with active involvement of academic researchers [39]. These factors explain why the expansion of CS platforms in developing countries might be limited by the availability of necessary infrastructures [39]. Secondly, this geographic pattern is consistent with the tradition of CS, which emerged in North America and then spread globally, primarily driven by some iconic platforms and surveys such as the Christmas Bird Count, eBird, and Project BudBurst [76]. Lastly, in regions with fewer papers using CS data, sharing biodiversity data remains difficult due to a lack of a tradition of open-access databases, but also to language barriers [39, 83]. A limitation of our study is that papers in languages other than English were not included in our review. These papers represent approximately 15% of papers within Scopus [84]. Thus, we acknowledge that restricting our review to English papers may have reduced our coverage of certain areas such as Arab countries, Latin America, or Asia [82]. In addition to language barriers and geographic location, national security concerns and economy also creates spatial variations in the coverage of global databases [82].

### Source of data

**Fig 4:**
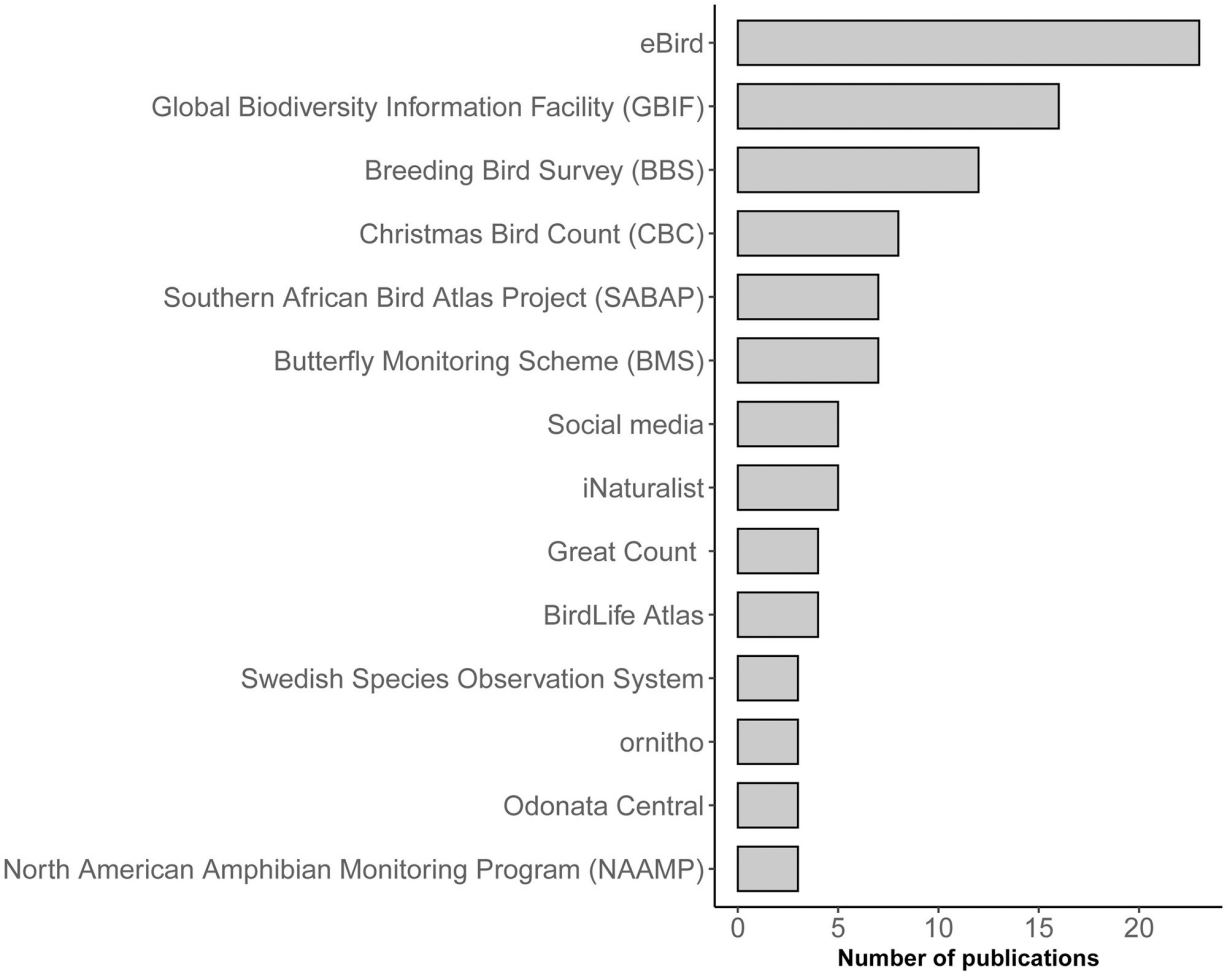
Sources of information used in the papers included in the literature review (n = 207). Only databases with three or more papers are shown. Great Count: world-wide surveys targeting birds and mammals.

A wide variety of sources were identified, most being taxa-specific (e.g. birds or flying insects), whereas platforms that included various taxa were used to a lesser extent. The main reason for the predominance of bird CS papers was the use of three widespread networks of birders: the eBird project, the Breeding Bird Survey (BBS), and the Southern African Bird Atlas Project (SABAP, Fig 4). For insects, the Butterfly Monitoring Scheme (BMS) was the most widely used. Even if GBIF was the second most used source of information, this portal aggregates global biodiversity information from a variety of sources [39], including other CS portals listed in Fig 4. Indeed, the major GBIF contributor is eBird [39, 82]. For that reason, the GBIF database cannot be dissociated from other sources of CS in Fig 4.

### Study scope

**Fig 5:**
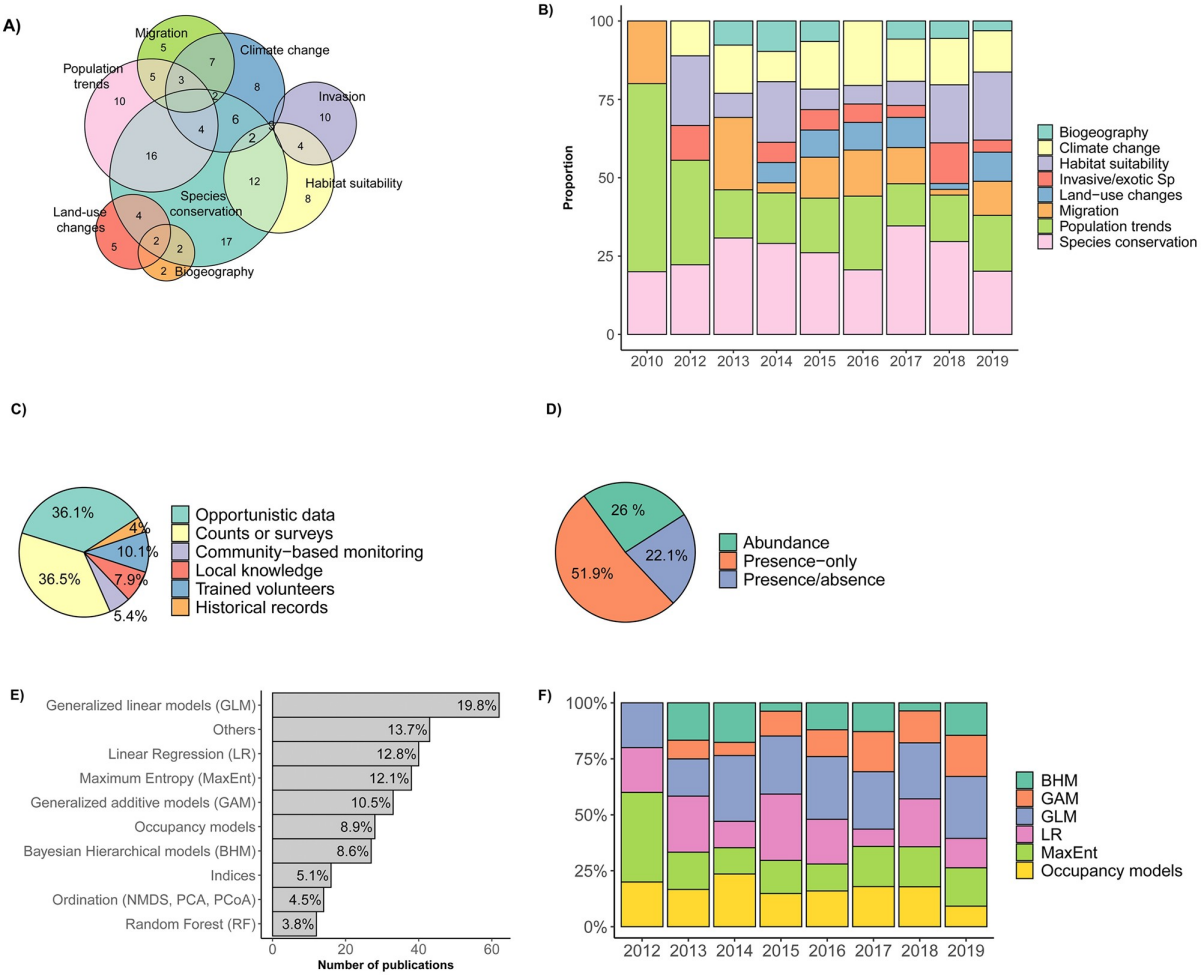
Papers that have analyzed species distribution models (SDMs) using citizen science (CS) data in our literature review illustrating (a) study scope illustrated by a Venn diagram (intersections containing a single study not shown); (b) study scope per year; (c) method of collecting citizen science data; (d) data type used; (e) statistical approach and (f) statistical approach per year. All details for the 207 papers are cited in S1 Table.

Although the scope and geographical coverage varied greatly among SDM papers using CS data (Fig 5a), most papers addressed issues related to species conservation (n = 95; 25.5%), followed by population trends (n = 67; 17.9%), habitat suitability (n = 56; 15%), and climate change (n = 52; 13.9%) (Fig 5a). Furthermore, species conservation was the central aim of these studies throughout the last decade, particularly in recent years (Fig 5b). The same pattern was observed for SDMs in tropical regions [1]. Most species conservation papers using CS documented species of conservation concern, rare species, or poorly-studied regions [34, 85].

### Method of collecting citizen science data

Several methods of collecting CS data were reported in the papers reviewed. The predominant method of collection consisted of count surveys (n = 101; 36.5%). In contrast to opportunistic data collection, count surveys differ in the structure of the methodology used, and may involve transect counts, point counts, or censuses. The second most used was opportunistic data collection which accounted for 36.1% (n = 100) of the papers (Fig 5c). With the growing popularity of online databases compiling occurrence data, the predominance of opportunistic data collection is not surprising [86]. Opportunistic data can be collected in many forms, including crowdsourcing databases or single random observations stored on online portals. Historical databases store information on past occurrence data usually collected opportunistically but generally found in naturalist diaries, letters or newspapers [87]. Our review found only 11 papers in the last decade that included historical databases (3.9% of the papers reviewed) (Fig 5c). Among other methods of collection, CS with trained volunteers only comprised a small proportion (n = 28; 10,1%) of the papers reviewed, highlighting that training volunteers is not a barrier to publishing. Nevertheless, projects with trained volunteers are more likely to be published than projects without training [60]. Data collection based on local knowledge (n = 22; 7,9%) and community-based monitoring (CBM) (n = 15; 5,4%) were rarely used (Fig 5c). Local and traditional knowledge of indigenous communities could improve SDMs. Indeed, by combining multiple types (or system) of knowledges, the collaboration between researchers and indigenous communities may help to expand the understanding of distribution boundaries, habitat and environmental associations, incorporate long-term observations, find local solutions for conservation actions and support the maintenance of local languages and culture. Additionally, engaging local participation may incorporate spatial guidance for gathering species records, increase the quantity of the data collected and expand the number of taxa covered by incorporating more observers [88, 89]. However, to fully benefit from this data collection method, researchers must be familiar with social science methods. Researchers may encounter difficulties in cross-cultural interactions, including language communication barriers and the reticence of the communities to share information about their environment [90]. Despite such difficulties, both scientist and communities can benefit from building on the interest and concerns of local community members when applying local knowledge and CBM [81]. Usually, the full potential of CBM programs is expressed when local communities participate actively during the entire scientific program, from the conceptual design and interpretation of results to the formulation of conclusions [88, 91]. Such cases have rarely occurred in the last decade for SDMs, probably because these programs are typically designed to monitor environmental factors rather than to collect species occurrence.

### Data type

Citizen science data usually consist of presence-only data because they are easier to collect and require less effort than any other type of data. In our literature review, 120 out of 207 (52%) papers used PO data, 60 (26%) used abundance data and 51 (22%) used presence-absence data (Fig 5d). Twenty-two papers used two types of data (see S1 Table) and one paper used all three data types [92]. Several authors have highlighted the limitations of presence-only data, which confound information about habitat preferences and availability, have a strong spatial bias with more effort in sampling certain areas than others, and ignore environmental conditions associated with species occurrence [19, 27, 93]. Despite such restrictions, prominent modelling approaches dealing with presence-only data in the SDM literature can provide a performance comparable to models with presence-absence data [90, 94]. Some of those techniques include MaxEnt [95–97] especially for rare species or remote areas with low data points, spatial point-process models [98–100], or generalized additive models (GAMs) [101]. Furthermore, when absence data is not available in presence-only models, an alternative approach to account for potential sampling bias is to simulate pseudo-absences by including background information about non-occupied environments [19, 53, 102]. This is usually achieved by treating random point locations as absences in the same numbers as the presence data set [103–105]. Methods that generate simulated pseudo-absences instead of presence-only are an open research field for SDMs [104, 106, 107], highlighted in our review for 27 (13%) papers. After the generation of pseudo-absences, both the presence and pseudo-absence data can be analyzed using standard analyses for presence-absence data [108, 109], which is appropriate for a wide range of SDMs [110–112]. Despite these recent advances in presence-only data, there were high proportions of presence-absence and abundance data in the papers reviewed (n = 111; 48% in total; Fig 5d). Presence-absence data allow the comparison of a species’ occupancy between different areas or time periods [27], but is generally less common in CS data. Recent developments of various occupancy model types, accounting for imperfect detection probability [93, 113, 114], contributed to the increasing use of presence-absence data to infer the spatial distribution of species. Hence, this development would explain the increasing use of PA data obtained from CS databases. Abundance (AB) data occurred in similar proportions to PA in the set of studies reviewed (n = 60, 26%). Information on the number of individuals is essential to detect changes in population sizes [27]. Presence-absence (PA) and AB data can both be obtained from checklists, point-counts, or transect surveys by volunteers [93].

### Statistical approach

The statistical approaches used in the papers reviewed were diverse, including linear regression approaches (LR; n = 40; 12.8%), maximum entropy (MaxEnt; n = 38, 12.4%), generalized linear models (GLM; n = 62; 19.8%), occupancy models (n = 28; 8.9%), and generalized additive models (GAM; n = 33; 10.5%; Fig 5e). Presence-only (PO) data were most frequently analyzed with MaxEnt (n = 36), whereas PA data were most frequently analyzed with occupancy models and GLM (n = 17 and 12, respectively). Abundance (AB) data were most often analyzed with GLM (n = 24). The proportion of use of the statistical approaches in the CS papers we reviewed did not seem to change between 2010 and 2019, with the exception of Bayesian hierarchical models (BHM) and GAMs appearing in papers from 2013 onward (Fig 5f).

### Multiple data sources

Of the 207 articles reviewed, 81 (39.1%) used multiple sources of data, merging data from CS recorded by the public with professional data collected by experts. In 29 of these cases, authors compared results of volunteers’ observations with those obtained by professional scientists and only three studies revealed mismatches, particularly for species abundance [51, 115, 116]. The integration of CS and professional data has been a growing trend in recent years [105, 117, 118] and shows promise to improve inferences and the predictive ability of models, as well as to fill knowledge gaps for under-studied areas or poorly studied species [105, 117–119]. This approach of combining data benefits from robust survey schemes and expands the geographic and taxonomic coverage using unstructured opportunistic schemes. However, integrating highly heterogeneous data types such as large unstructured presence-only data and standardized abundance surveys, is still challenging for modelling purposes.

## Conclusions

In this review, we examined the trends and information gaps in the use of citizen science (CS) data for species distribution models (SDMs) in peer-reviewed papers over the last decade. Citizen science already makes substantial contributions to the field of SDMs and this trend will probably continue. We presented examples of the use of CS and highlighted recommendations to motivate further research, such as combining multiple data sources and promoting local and traditional knowledge.

The reviewed citizen science papers considered a wide range of taxa, regions, and countries, from numerous biomes and landscape forms. However, taxonomic and geographic unevenness of CS projects for SDMs still remain [39, 48, 120]. It is imperative to better cover a wide range of taxonomic diversity to optimize the use of SDMs for species conservation. Accounting for these disparities in CS is crucial to adequately cover spatial and temporal scales, and strategically deploying formal surveys in areas or for species not covered by volunteers can be a key to better predict species distribution. Despite its huge contribution, the potential of citizen science can be maximized only if its value is recognized and data are analyzed rigorously. Therefore, we strongly encourage that researchers use as well as actively contribute to citizen science because they might have a major impact over the entire community of observers. The active participation of researchers in citizen science platforms (e.g. validating species identifications) can not only increase the amount of accessible data, but also increase the interest of local participants in countries where little information is currently available on the distribution of certain species.

## Supporting information

S1 Table Methodologies of 207 papers published from 2010 to 17 October 2019 that used citizen science data to model species distribution resulting from the above described search protocol in the Scopus database. (DOCX)

S1 Checklist (DOC)
