# Supplementary material for: Trends and gaps in the use of citizen science derived data as input for species distribution models: A quantitative review
Source: PLoS One. 2021 Mar 11;16(3):e0234587. doi: 10.1371/journal.pone.0234587 (PMC7951830; doi:10.1371/journal.pone.0234587)
Supplement: S1 Table — (DOCX) [file pone.0234587.s001.docx]

**S1 Table**. Summary of methodologies of 207 articles published from 2010 to 17 October 2019 that have used citizen science data to model species distribution.

| **Reference** | **Year** | **Taxa** | **Country** | **Region** | **Scope** | **Method of collecting CS data** | **Data type** | **Statistical approach** | **Multiple data sources** |
| --- | --- | --- | --- | --- | --- | --- | --- | --- | --- |
| Aizpurua *et al* (1) | 2015 | bird | Belgium | W-Eur | bio | OP | PO | MaxEnt | no |
| Allen *et al* (2) | 2019 | bird | Netherlands | W-Eur | mig, pop | OP | PO | CMR | yes |
| Allen *et al* (3) | 2019 | bird | Netherlands | W-Eur | mig, pop | OP | PO | CMR | no |
| Allen *et al* (4) | 2019 | fish | USA | N-Ame | cli, hab, pop | TR | PO | GLMM, LM | no |
| Alves *et al* (5) | 2019 | bird | Iceland | W-Eur | cli, mig, pop | OP | PO | GLMM | no |
| Andrew *et al* (6) | 2019 | fungus | NA | Europe | cli, lan | OP, HI | PO | GAMM | yes |
| Arthur *et al* (7) | 2014 | mammal | France | W-Eur | hab | TR | PO | GLM, GAM | no |
| Ashcroft *et al* (8) | 2012 | hymenoptera | Australia | Ocean | hab, inv | OP, TR | PO | MaxEnt | yes |
| Baker *et al* (9) | 2019 | bird | Australia | Ocean | cli, con, pop | CO | AB | Occupancy | no |
| Balestrieri *et al* (10) | 2019 | mam | Italy | W-Eur | lan | OP | PO | Other | yes |
| Barrows *et al.* (11) | 2016 | reptile | NA | N-Ame | cli | TR | AB | Ordin | yes |
| Bauer et al (12) | 2019 | spider | Germany, Ecuador | W-Eur, S-Ame | inv | OP | PO | Ordin, MaxEnt | yes |
| Beale et al (13) | 2019 | fish | Indonesia | Asia | con, cli | OP, LEK | AB | CMR | no |
| Belt & Krausman (14) | 2012 | mammal | USA | N-Ame | pop | TR | AB | N-mixt | yes |
| Blanc *et al.* (15) | 2014 | mammal | France | W-Eur | con, pop | CO | PA, AB | CMR, BHM, Occupancy | yes |
| Bled *et al.* (16) | 2013 | bird | S-Africa | Africa | bio, cli | CO | PA | BHM, GAM | no |
| Bonnet-Lebrun et al (17) | 2019 | mammal | Greece | W-Eur | con, hab | OP | PO | GAM | no |
| Botella *et al.* (18) | 2018 | plant | France | W-Eur | inv | OP | PO | MaxEnt | no |
| Boyle & Sigel (19) | 2015 | bird | Costa Rica | C-Ame | con, cli, hab, pop | CO | PA | GLM, indices | no |
| Bradsworth *et al.* (20) | 2017 | bird | Australia | Ocean | con, hab | CO, CBM | PO | MaxEnt, indices | no |
| Bradter *et al.* (21) | 2018 | bird | Sweden | W-Eur | con, cli, hab, inv | OP | PO | MaxEnt, BHM, LR | yes |
| Breininger et al (22) | 2019 | reptile | USA | N-Ame | con, hab | CO | AB | BHM, N-mixt, occupancy | no |
| Bried & Siepielski (23) | 2018 | odonata | USA | N-Ame | bio | OP, CO | PO, PA | Ordin | no |
| Brommer *et al.* (24) | 2017 | mammal | Finland | W-Eur | bio, inv, pop | LEK | AB | N-mixt, BHM | no |
| Broms *et al.* (25) | 2014 | bird | S-Africa | Africa | hab | CO, CBM | PA | BHM, occupancy | no |
| Buldrini *et al.* (26) | 2015 | plant | Italy | W-Eur | con, inv, pop | OP | PO | indices | no |
| Butler *et al.* (27) | 2016 | reptile | USA, Mexico | N-Ame | cli | OP | PO | MaxEnt | yes |
| Camacho (28) | 2016 | bird | Chile, Peru | S-Ame | con, pop | OP | PO | GLM | no |
| Campbell & Engelbrecht (29) | 2018 | spider | S-Africa | Africa | con | OP | PO | BRT | yes |
| Cantú‐Salazar & Gaston (30) | 2013 | amphibian, bird, mammal | NA | America | con | CO | PO | MaxEnt | yes |
| Cerrano *et al.* (31) | 2017 | mollusca | NA | Europe | con, cli | CO, TR | PA, AB | Other | no |
| César de Sá et al (32) | 2019 | plant | Portugal | W-Eur | inv, hab | OP | PO | GLM, GAM, RF, MaxEnt | yes |
| Champion *et al.* (33) | 2018 | fish | Australia | Ocean | cli, hab | LEK | PO | GAMM | no |
| Clare et al (34) | 2019 | mammal | USA | N-Ame | NA | TR | PO | GLM, BHM | no |
| Colino-Rabanal & Peris (35) | 2016 | mammal | Spain | W-Eur | NA | OP | PO | LR | yes |
| Collins *et al.* (36) | 2017 | odonata, lepidoptera | USA | N-Ame | hab | CO | PA | MaxEnt | no |
| Coxen *et al.* (37) | 2017 | bird | USA | N-Ame | cli, mig | CO | PO | MaxEnt | yes |
| Crall *et al.* (38) | 2015 | plant | USA | N-Ame | hab, inv | TR | PO | MaxEnt | yes |
| Crewe et al (39) | 2019 | lepidoptera | Canada | N-Ame | lan, mig, pop | OP, CO CBM | AB, PO | LM | yes |
| Croft et al (40) | 2019 | mammal | UK | W-Eur | con | OP | PO | BHM | no |
| Crone et al (41) | 2019 | lepidoptera | NA | N-Ame | con, cli, hab, lan, pop | CO | AB | LM | no |
| Crum *et al.* (42) | 2017 | mammal | USA | N-Ame | con, lan | CO, LEK | PA, AB | Occupancy | no |
| Davis *et al.* (43) | 2014 | bird | USA | N-Ame | hab | CO | PO | RF | no |
| De Coster *et al.* (44) | 2015 | bird | Belgium | W-Eur | lan, pop | CO | AB | GLM, LR | no |
| de Medeiros *et al.* (45) | 2018 | hymenoptera | Iberian peninsula | W-Eur | inv | OP | PO | MaxEnt | yes |
| De Rock et al (46) | 2019 | mammal | Namibia | Africa | con, hab | OP | PO | MaxEnt | yes |
| de Sá Dechoum *et al.* (47) | 2019 | plant | Brazil | S-Ame | hab, inv | CO | PA | RF, BRT, GLM | no |
| De Solan *et al.* (48) | 2018 | reptile | France | W-Eur | bio, lan | OP | PO | Ordin, LR | yes |
| Dennhardt *et al.* (49) | 2015 | bird | USA | N-Ame | con, mig | CO | PO | CMR | yes |
| Dennis *et al.* (50) | 2019 | lepidoptera | Scotland | W-Eur | hab, land, pop | OP, CO | AB, PO | Occupancy, GLM | no |
| Dennis *et al.* (51) | 2017 | lepidoptera | UK | W-Eur | pop | CO | AB | GLM | yes |
| Derville *et al.* (52) | 2018 | mammal | New Caledonia | Ocean | con, hab | OP, TR | PO | BRT, GLM, MaxEnt, GAM | yes |
| Desaegher et al (53) | 2019 | plant | France | W-Eur | land | CO | PA | LM, GLMM | no |
| Deutsch *et al.* (54) | 2017 | amphibian | Argentina | S-Ame | con | CO, LEK | PO | Other | yes |
| Dilts *et al* (55) | 2019 | lepidoptera, plant | USA | N-Ame | cli, con, hab, mig, pop | OP | PO | MaxEnt | yes |
| Dissanayake et al (56) | 2019 | mammal | Australia | Ocean | land, pop | OP | PO | Other | no |
| Dörler *et al.* (57) | 2018 | mollusca | Spain | W-Eur | inv | TR | AB, PO | GLM | no |
| Droz *et al.* (58) | 2019 | bird | Switzerland | W-Eur | con, hab, lan | OP | PO | GLM, ordin, GAM, Maxent | yes |
| Dunn (59) | 2019 | bird | NA | N-Ame | mig, pop | CO | AB | GAM, GLM | yes |
| Edgar *et al.* (60) | 2018 | fish | Australia | Ocean | cli, pop | CO, LEK | AB | GLM | yes |
| Evangelista *et al.* (61) | 2018 | mammal | Somaliland | Africa | con, hab | LEK | PO | BRT, MaxEnt | no |
| Evans et al (62) | 2019 | coleptera | NA | N-Ame | cli | CO | AB | LR | no |
| Fabrizzio (63) | 2019 | mammal | Italy | W-Eur | con, hab | OP | PO | MaxEnt | no |
| Fink *et al.* (64) | 2010 | bird | USA | N-Ame | mig | CO | PA | STEM | no |
| Flaherty (65) | 2019 | mammal | Ireland | W-Eur | inv, lan | OP | PO | Other | yes |
| Flesch & Belt (66) | 2017 | mammal | USA | N-Ame | NA | TR | PO | Occupancy | yes |
| Fournier *et al.* (67) | 2017 | bird | NA | N-Ame | con, mig | LEK | PO | Other | no |
| Gange (68) | 2019 | fungus | UK, Switzerland | W-Eur | bio, con, hab, pop | OP | AB | GAM, indices | yes |
| Girado-Beltrán *et al.* (69) | 2015 | plant | Spain | W-Eur | inv | OP, HI | PO | GLMM, LR | yes |
| Girardello (70) | 2019 | lepidoptera | NA | Global | con | OP | PO | BHM, GLM | no |
| Giroux *et al.* (71) | 2016 | bird | Canada | N-Ame | cli, mig, pop | CO | PA | LR | no |
| Goodenough (72) | 2014 | bird | UK | W-Eur | con, hab, mig | TR | PO | LR | no |
| Goodwin *et al.* (73) | 2017 | mammal | UK | W-Eur | con, pop | CO, TR, CBM | PO, AB | GAM, GLM | No |
| Gorta (74) | 2019 | bird | Australia | Ocean | con, cli, pop | CO | AB, PA | GAM, GLM | no |
| Goswami *et al.* (75) | 2015 | mammal | India | Asia | lan | TR | PO | Occupancy | no |
| Grüss *et al.* (76) | 2019 | fish | NA | Gulf of Mexico | NA | CO | PO | GLM, GAM | yes |
| Hackworth (77) | 2019 | bird | USA | N-Ame | hab, lan, pop | OP | PO | Other | yes |
| Hahn *et al.* (78) | 2016 | hemiptera | USA | N-Ame | NA | OP | PO | LR | no |
| Hallworth *et al.* (79) | 2015 | bird | NA | N-Ame | bio, con, mig | CO | AB | N-mixt | no |
| Hansen *et al.* (80) | 2015 | bird | Australia | Ocean | mig, pop | CO | AB | LR | no |
| Hart *et al.* (81) | 2018 | hymenoptera | UK | W-Eur | bio | OP | PO | GLMM | no |
| Hertzog *et al.* (82) | 2014 | coleoptera | France, Iberian peninsula | W-Eur | lan, pop | OP, HI | PO | BRT, MaxEnt | no |
| Hieb *et al.* (83) | 2017 | mammal | NA | Gulf of Mexico | con, lan | OP, LEK | PO | LR | no |
| Higa *et al.* (84) | 2015 | bird | Japan | Asia | NA | OP | PO, PA | LR, occupancy | yes |
| Hill et Lloyd (85) | 2017 | bird | USA, Canada | N-Ame | con, cli, pop | CO | AB | N-mixt | no |
| Horns *et al.* (86) | 2018 | bird | NA | N-Ame | con, pop | OP, CO | PA | LR | no |
| Horton (87) | 2019 | bird | NA | Gulf of Mexico | cli, mig | CO | AB | RF, STEM, GAMM, GAM | no |
| Hosseini (88) | 2019 | mammal | Iran, Turmekistan, Afganistan | Asia | con, hab | OP, LEK | PO | MaxEnt | yes |
| Howes (89) | 2019 | bird | NA | Africa | hab, mig, pop | CO | AB | GLMM | yes |
| Hugo & Altwegg (90) | 2017 | bird | S-Africa, Lesotho, Swaziland | Africa | NA | OP, TR | PO | LR, ordin, GLMM | no |
| Humphreys (91) | 2019 | bird | USA | N-Ame | bio, con, hab, mig | CO | PA, AB | BHM | no |
| Ivanova (92) | 2019 | bird | S-Africa | Africa | inv, hab, pop | CO | PO, AB | GLM | no |
| Jackson *et al.* (93) | 2015 | bird | Canada | N-Ame | cli, con, hab | OP, CO, LEK | PO | RF, BRT, GLM, MaxEnt, GAM | yes |
| Jiguet *et al.* (94) | 2012 | bird | NA | Europe | cli, con, pop | CO, TR, CBM | AB | CMR, LR | no |
| Jiménez-Valverde (95) | 2019 | spider | Spain | W-Eur | con | OP | PO | GAM, LR | yes |
| Johnston *et al.* (96) | 2018 | bird | NA | NA | pop | CO | PA | GAM, Occupancy | no |
| Jones *et al.* (97) | 2013 | mammal | NA | Europe | NA | OP | PO | MaxEnt | No |
| Kamp *et al.* (98) | 2016 | bird | Denmark | E-Eur | con, pop | CO, HI | AB | Occupancy, GLMM | yes |
| Kasahara & Koyama(99) | 2010 | bird | Japan | Asia | pop | CO, CBM | AB | GLM, indices | no |
| Kery *et al.* (100) | 2010 | odonata | Switzerland | W-Eur | NA | CO | PA | GLM, Occupancy | no |
| Kery *et al.* (101) | 2010 | bird | Switzerland | W-Eur | pop | OP | PA | Occupancy | yes |
| Koparde (102) | 2019 | bird | NA | Eurasia | con, bio, cli, hab | OP | PO | MaxEnt | Yes |
| Kreling (103) | 2019 | mammal | USA | N-Ame | lan | OP | PO | LR | no |
| Krolikowska *et al.* (104) | 2018 | bird | Poland | W-Eur | con, hab, pop | TR | PA | RF | yes |
| La Sorte *et al.* (105) | 2017 | bird | NA | C-Ame | cli, lan, mig, pop | CO | PA | BRT, STEM, GAMM | no |
| La Sorte *et al.* (106) | 2019 | bird | NA | N-Ame | cli, mig | CO | AB | GAMM, STEM, GLM | no |
| La Sorte *et al.* (107) | 2015 | bird | USA | N-Ame | con, mig, cli | CO | PA | STEM, GAMM | no |
| La Sorte *et al.* (108) | 2016 | bird | NA | N-Ame | cli, mig | CO | PA | NA | no |
| Laughlin *et al.* (109) | 2016 | bird | USA | N-Ame | mig | CO | PA | STEM, GAM, LR | yes |
| Laughlin *et al.* (110) | 2013 | bird | NA | N-Ame | hab, mig | CO | PA | LR | yes |
| Lees & Martin (111) | 2015 | bird | Brazil | S-Ame | bio, con, lan | OP, HI | PO | LR | yes |
| Lin *et al.* (112) | 2015 | lepidoptera | Taiwan | Asia | NA | OP | PO | MaxEnt | yes |
| Long (113) | 2019 | bird | USA | N-Ame | con, hab, mig | CO | PO | Other | no |
| Looney (114) | 2019 | hymenoptera | NA | N-Ame | hab | OP | PO | MaxEnt | no |
| Louvrier *et al.* (115) | 2019 | mammal | NA | W-Eur | con | OP, LEK | PO | Occupancy | yes |
| Luigi Nimis *et al.* (116) | 2018 | plant | Italy | W-Eur | inv, lan, mig | OP | PO | Ordin | yes |
| Luizza *et al.* (117) | 2016 | plant | Ethiopia | Africa | hab, inv | CBM | PO | MaxEnt, indices | yes |
| Lyon (118) | 2019 | fish | Australia | Ocean | hab, pop | LEK | AB | CMR, BHM | yes |
| MacPhail (119) | 2019 | hymenoptera | Canada | N-Ame | con, pop | OP, CO, HI | PO, AB | LR | yes |
| Mair *et al.* (120) | 2017 | fungus | Sweden | W-Eur | con, lan | TR, CO | PO | GLM, MaxEnt, Occupancy | no |
| Marsh *et al.* (121) | 2019 | amph | NA | N-Ame | NA | CO, TR | PA | LR | no |
| Marsh *et al.* (122) | 2017 | amph | USA | N-Ame | bio, con, lan | CO | PA | GLM | no |
| Martin (123) | 2019 | bird | Iberian Peninsula | W-Eur | mig, pop | CO, TR | PA, AB | LR | yes |
| Martin (124) | 2019 | plant | France | W-Eur | cli, hab | CO, CBM | PA, AB | BHM | no |
| Marx & Quillfeldt (125) | 2018 | bird | Germany | W-Eur | con, hab | CO, OP | PO, PA | BioClim, RF, CART, GLM | no |
| Mason *et al.* (126) | 2018 | lepidotpera | UK | W-Eur | bio, con | CO, OP | AB | LR, indices | no |
| Massimino *et al.* (127) | 2018 | mammal | UK | W-Eur | con, pop | OP | AB | GAM | no |
| McCoshum *et al.* (128) | 2016 | lepidotpera | USA | N-Ame | con, mig | OP | PO | MaxEnt | yes |
| McDuffie (129) | 2019 | bird | USA | N-Ame | pop | OP | PO, AB | GLMM | no |
| Meehan (130) | 2019 | bird | NA | N-Ame | pop | CO | AB | BHM | no |
| Melero *et al.* (131) | 2016 | lepidotpera | Spain | W-Eur | pop | CO | AB | BHM, GAMM, GLMM, GLM | no |
| Miller *et al.* (132) | 2016 | bird | USA | N-Ame | con, hab, lan, pop | CO, TR | AB | N-mixt, Occupancy | no |
| Mims *et al.* (133) | 2018 | amphibian, reptile, fish | USA | N-Ame | cli, con | OP | PO | RF, ordin, CART, indices | yes |
| Mingozzi *et al.* (134) | 2013 | bird | Italy | W-Eur | cli, mig | OP | AB | LR | no |
| Mohanty & Measey (135) | 2018 | amphibian | India | Asia | inv | CO, LEK | PA | Occupancy | no |
| Mohanty *et al.* (136) | 2018 | bird, mollusca | India | Asia | inv | LEK | PA | Occupancy | no |
| Molinari‐Jobin *et al.* (137) | 2018 | mammal | NA | W-Eur | con | OP, TR | PO | Occupancy | no |
| Monsarrat *et al.* (138) | 2018 | mammal | S-Africa | Africa | con | HI | PO | Indices | no |
| Morii & Nakano (139) | 2017 | mollusca | Japan | Asia | inv | OP, LEK | PO | NA | no |
| Mosnier (140) | 2019 | reptile | Canada | N-Ame | cli, hab | OP, CO, LEK | PO | GAM | yes |
| Musilová *et al.* (141) | 2018 | bird | Czech Rep | E-Eur | cli, hab, pop | CO | AB | LR | no |
| Musilová *et al.* (142) | 2018 | bird | Czech Rep | E-Eur | con, hab, pop | CO | AB | LR, GLMM | no |
| Nagy *et al.* (143) | 2012 | bird | USA | N-Ame | hab | CO, TR | PA | Occupancy | yes |
| Newson *et al.* (144) | 2016 | bird | UK | W-Eur | con, cli, pop | CO | PA, AB | GAM | yes |
| Pace (145) | 2019 | mammal | Italy | W-Eur | con, hab | OP, CO | PO | MaxEnt, GLM | yes |
| Pacifici (146) | 2019 | bird | USA | N-Ame | NA | CO | PA | Occupancy | yes |
| Pagel *et al.* (147) | 2014 | lepidoptera | UK | W-Eur | bio, con, pop | OP, CO | PO, PA, AB | BHM | no |
| Paukkunen *et al.* (148) | 2018 | hymenoptera | Finland | W-Eur | con, hab, pop | OP | PO, AB | Other | yes |
| Peach *et al.* (149) | 2017 | bird | USA | N-Ame | con, pop | CO | PO | Occupancy, BHM | no |
| Penã-Aguilera (150) | 2019 | spider | Iberian peninsula | W-Eur | cli | OP | PO | Ordin | yes |
| Péron & Altwegg (151) | 2015 | bird | S-Africa | Africa | NA | CO | PA | Occupancy, ordin | no |
| Péron & Altwegg (152) | 2015 | bird | S-Africa, Lesotho, Swaziland | Africa | cli, con, land, pop | CO | PA | Occupancy | no |
| Pescott *et al.* (153) | 2015 | lichen, bryophyte, lepidoptera | UK | W-Eur | NA | OP | PO | LR | no |
| Phillips *et al.* (154) | 2017 | fish | NA | Global | bio, con, hab, mig | OP | PO | MARS, RF, CART, GLM, GAM | no |
| Pillay *et al.* (155) | 2014 | mammal, bird, reptile | India | Asia | bio, con | LEK | PA | Occupancy | yes |
| Plummer *et al.* (156) | 2015 | bird | UK | W-Eur | cli, con, mig | CO | PA | GLMM | no |
| Prodon *et al.* (157) | 2017 | reptile, amphibian | France | W-Eur | cli, mig | OP | PO | GLM, GAM | no |
| Puan (158) | 2019 | bird | Malasya | Asia | lan | CO | AB | GLMM | no |
| Ralston *et al.* (159) | 2015 | bird | USA | N-Ame | con, pop | CO | PA | LR | no |
| Rapacciuolo *et al.* (160) | 2017 | odonata | USA | N-Ame | con, pop | OP, HI | PO | BHM | yes |
| Reed *et al.* (161) | 2017 | mammal | USA | N-Ame | hab | OP | PA | GLM, LR | yes |
| Ribeiro (162) | 2019 | bird | Portugal | W-Eur | NA | OP | PO | GLM | no |
| Richardson *et al.* (163) | 2015 | plant | New Zealand | Ocean | con | OP | PO | Ordin, GLM | yes |
| Robinson *et al.* (164) | 2018 | bird | USA | N-Ame | con | CO | PO | RF, CART | no |
| Robinson *et al.* (165) | 2014 | bird | UK | W-Eur | con, pop | CO | AB | GLM | no |
| Rodewald (166) | 2019 | bird | Colombia, Ecuator, Peru | S-Ame | con, mig, pop | CO | AB | STEM | no |
| Rodhouse *et al.* (167) | 2015 | mammal | USA | N-Ame | con, pop | OP, CO | PO | BHM | yes |
| Ruete *et al.* (168) | 2017 | bird | Sweden | W-Eur | mig | OP | PO | BHM | no |
| Ruiz‐Gutierrez *et al.* (169) | 2016 | amphibian | NA | N-Ame | NA | CO | PA | BHM | no |
| Rutten (170) | 2019 | mammal | Belgium | W-Eur | hab | OP, LEK | PO | MaxEnt | no |
| Santika *et al.* (171) | 2014 | mammal | Australia | Ocean | cli, con, lan | OP | PO | Occupancy | no |
| Sardà-Palomera *et al.* (172) | 2012 | bird | Spain | W-Eur | con | OP, CO, CBM | PO, PA | MaxEnt | no |
| Sequeira *et al.* (173) | 2014 | mammal | Australia | Ocean | cli, con, hab | CO | PA | GLMM, GLM | no |
| Sillero *et al.* (174) | 2014 | amphibian, reptile | NA | Europe | bio, con, cli | OP, CO, CBM | PO | Indices | yes |
| Smale (175) | 2019 | plant | UK | W-Eur | con, hab | TR, CO | PA, AB | GLMM, Ordin | no |
| Snäll *et al.* (176) | 2011 | bird | Sweden | W-Eur | NA | CO | PO | BHM, indices | yes |
| Soroye *et al.* (177) | 2018 | lepidoptera | Canada | N-Ame | cli | OP | PO | LR | yes |
| Soykan *et al.* (178) | 2016 | bird | NA | N-Ame | cli, con, lan, pop | CO | AB | GLM, BHM | yes |
| Stefanescu *et al.* (179) | 2013 | lepidoptera | NA | Western Paleoartic | mig | CO | AB | GLM | yes |
| Summer (180) | 2019 | hymenoptera | UK | W-Eur | NA | OP, TR | PO | GLM, GAM | yes |
| Supp *et al.* (181) | 2015 | bird | NA | N-Ame | cli, mig | CO | PO | GAMM | no |
| Sweet (182) | 2019 | plant | USA | N-Ame | cli, con | CO | PO | MaxEnt | yes |
| Szabo *et al.* (183) | 2010 | bird | Australia | Ocean | con, pop | CO, HI | PO | LR | no |
| Termaat (184) | 2019 | odonata | NA | W-Eur | cli | OP | PO | Occupancy | no |
| Thorson *et al.* (185) | 2014 | fish | USA | N-Ame | hab, pop | OP, LEK | AB | GLMM | no |
| Tiago *et al.* (186) | 2017 | amphibian, reptile | Portugal | W-Eur | cli | OP, CO, CBM | PO | GLMM, GAM | yes |
| Titeux *et al.* (187) | 2017 | lepidoptera | NA | Europe | cli, con | OP, CBM | PO | GAM | no |
| Todd *et al.* (188) | 2016 | amphibian, reptile | USA | N-Ame | con, lan | OP, CBM | PO | Other | no |
| Tonachella *et al.* (189) | 2012 | mammal | USA | N-Ame | pop | CO | AB | GLMM | no |
| Tulloch *et al.* (190) | 2013 | bird | Australia | Ocean | con | CO, CBM | PO | GLM, LR | yes |
| Tye *et al.* (191) | 2017 | mammal | USA | N-Ame | con | OP | PO | MaxEnt | yes |
| van Strien *et al.* (192) | 2013 | odonata | NA | Europe | con, pop | OP | PA | Occupancy, BHM | yes |
| van Strien *et al.* (193) | 2013 | odonata, lepidoptera | Netherlands | W-Eur | con, pop | OP | PA | Occupancy | no |
| van Strien *et al.* (194) | 2019 | lepidoptera | Netherlands | W-Eur | hab, pop | OP, HI, CO | PO | LR | yes |
| Vantieghem *et al.* (195) | 2017 | lepidoptera | Belgium | W-Eur | con | OP | PO | GLM, MaxEnt, GAM | yes |
| Veran *et al.* (196) | 2016 | coleoptera | France | W-Eur | inv | OP | PA | BRT, Occupancy | no |
| Villeneuve (197) | 2019 | mollusca | Dominica Rep | C-Ame | mig | TR | PO | CMR, LR | no |
| White *et al.* (198) | 2015 | fish | Costa Rica | C-Ame | pop | CO | AB | GLMM | no |
| Widenfalk *et al.* (199) | 2014 | lepidoptera, hemiptera | Sweden | W-Eur | con, inv | OP | PO, AB | MaxEnt, indices | no |
| Williams *et al.* (200) | 2016 | bird | Australia | Ocean | con, pop | CO | AB | LR, GLM | no |
| Wilson *et al.* (201) | 2015 | lepidoptera | UK | W-Eur | cli | CO, TR | AB | LR | no |
| Wine *et al.* (202) | 2015 | mammal | USA | N-Ame | inv | OP | PO | LR | no |
| Yue *et al.* (203) | 2019 | reptile | Taiwan | Asia | con, hab | OP | PO | MaxEnt | no |
| Zapponi *et al.* (204) | 2017 | coleoptera | Italy | W-Eur | cli, con | CBM, OP | PO | Other | yes |
| Zeng *et al.* (205) | 2018 | bird | China | Asia | con | TR | PA | RF | no |
| Zhang & Vincent (206) | 2017 | fish | China | Asia | con | LEK | PO | MaxEnt | yes |
| Zub *et al.* (207) | 2018 | mammal | NA | Europe | cli, con, hab | OP | PA | GLMM | no |

Region where the data were taken

*W-Eur:* Western Europe; *E-Eur:* Eastern Europe; *N-Ame:* North America; *S-Ame:* South America; *C-Ame:* Central America.

Scope or central objective of the study focus (if appearing in the title, abstract, or keywords)

*Con:* species conservation; *cli:* climate change, *hab:* habitat suitability; *inv*: invasive species; *lan*: land-use changes; *pop*: population trends, *mig:* migration; *bio*: biogeography.

Method of collecting citizen science data:

*CO*: counts or surveys (checklist, census, or surveys using a standardized method); *OP*: opportunistic data (single records collected randomly); *LEK*: local ecological knowledge (programs in collaboration with indigenous communities); *TR*: trained volunteers (some training is provided by experts before the collection of data); *CBM*: community-based monitoring (projects involving permanent collaboration of experts); *HIS*: historical records (past occurrence information compiled from historic documents or data entered retrospectively by observers or private collections)

Data type (nature of the occurrence data analysed)

*PO*: presence-only; *PA*: presence-absence; *AB*: abundance.

Statistical approach (model type used to link occurrence data with environmental variables based on [208]).

*Indices*: Indices of richness, abundance and others; *LR*: Multiple regression approaches; *GLM*: Generalized linear models; *MaxEnt*: maximum-entropy; *Ordin*: Ordination (NMDS, PCA, PCoA, PERMANOVA); *GAM*: Generalized Additive Models; *BHM*: Bayesian Hierarchical models; *RF*: Random Forest; *GLMM*: Generalized Linear mixed models; *BRT*: Boosted Regression trees; *N-mix*: N-mixture models; *GAMM*: Generalized additive mixed modelling; *CART*: classification and regression trees; *CMR*: Capture-mark-recapture models; *STEM*: The spatiotemporal exploratory model; *MARS*: Multivariate adaptive regression splines; *NN*: Neural networks; *MET*: mixed-effects trees; *GWR*; Geographically-weighted regression; *BioClim*: bioclimate analysis and prediction system.

References

1. Aizpurua O, Paquet JY, Brotons L, Titeux N. Optimising long‐term monitoring projects for species distribution modelling: how atlas data may help. Ecography. 2015; 38: 29-40. <https://doi.org/10.1111/ecog.00749>

2. Allen AM, Ens BJ, Van de Pol M, Van der Jeugd H, Frauendorf M, Oosterbeek K, et al. Seasonal survival and migratory connectivity of the Eurasian Oystercatcher revealed by citizen science. The Auk: Ornithological Advances. 2019; 136: uky001. <https://doi.org/10.1093/auk/uky001>

3. Allen AM, Ens BJ, van de Pol M, van der Jeugd H, Frauendorf M, van der Kolk H-J, et al. Colour-ring wear and loss effects in citizen science mark-resighting studies. Avian Research. 2019; 10: 11. <https://doi.org/10.1186/s40657-019-0151-z>

4. Allen DC, Kopp DA, Costigan KH, Datry T, Hugueny B, Turner DS, et al. Citizen scientists document long-term streamflow declines in intermittent rivers of the desert southwest, USA. Freshwater Science. 2019; 38: 244-256. <https://doi.org/10.1086/701483>

5. Alves JA, Gunnarsson TG, Sutherland WJ, Potts PM, Gill JA. Linking warming effects on phenology, demography, and range expansion in a migratory bird population. Ecology and evolution. 2019; 9: 2365-2375. <https://doi.org/10.1002/ece3.4746>

6. Andrew C, Büntgen U, Egli S, Senn‐Irlet B, Grytnes JA, Heilmann‐Clausen J, et al. Open‐source data reveal how collections‐based fungal diversity is sensitive to global change. Applications in plant sciences. 2019; 7. <https://doi.org/10.1002/aps3.1227>

7. Arthur L, Lemaire M, Dufrêne L, Viol IL, Julien JF, Kerbiriou C. Understanding bat-habitat associations and the effects of monitoring on long-term roost success using a volunteer dataset. Acta chiropterologica. 2014; 16: 397-411. <https://doi.org/10.3161/150811014x687350>

8. Ashcroft MB, Gollan JR, Batley M. Combining citizen science, bioclimatic envelope models and observed habitat preferences to determine the distribution of an inconspicuous, recently detected introduced bee (Halictus smaragdulus Vachal Hymenoptera: Halictidae) in Australia. Biological Invasions. 2012; 14: 515-527. <https://doi.org/10.1007/s10530-011-0092-x>

9. Baker D, Clarke R, McGeoch M. The power to detect regional declines in common bird populations using continental monitoring data. Ecological Applications. 2019: e01918. <https://doi.org/10.1002/eap.1918>

10. Balestrieri A, Mori E, Menchetti M, Ruiz‐González A, Milanesi P. Far from the madding crowd: Tolerance toward human disturbance shapes distribution and connectivity patterns of closely related Martes spp. Population Ecology. 2019. <https://doi.org/10.1002/1438-390x.12001>

11. Barrows CW, Hoines J, Vamstad MS, Murphy-Mariscal M, Lalumiere K, Heintz J. Using citizen scientists to assess climate change shifts in desert reptile communities. Biological conservation. 2016; 195: 82-88. <https://doi.org/10.1016/j.biocon.2015.12.027>

12. Bauer T, Feldmeier S, Krehenwinkel H, Wieczorrek C, Reiser N, Breitling R. Steatoda nobilis, a false widow on the rise: a synthesis of past and current distribution trends. NeoBiota. 2019; 42: 19. <https://doi.org/10.3897/neobiota.42.31582>

13. Beale CS, Stewart JD, Setyawan E, Sianipar AB, Erdmann MV. Population dynamics of oceanic manta rays (Mobula birostris) in the Raja Ampat Archipelago, West Papua, Indonesia, and the impacts of the El Niño–Southern Oscillation on their movement ecology. Diversity and Distributions. 2019; 25: 1472-1487. <https://doi.org/10.1111/ddi.12962>

14. Belt JJ, Krausman PR. Evaluating population estimates of mountain goats based on citizen science. Wildlife Society Bulletin. 2012; 36: 264-276. <https://doi.org/10.1002/wsb.139>

15. Blanc L, Marboutin E, Gatti S, Zimmermann F, Gimenez O. Improving abundance estimation by combining capture–recapture and occupancy data: example with a large carnivore. Journal of applied ecology. 2014; 51: 1733-1749. <https://doi.org/10.1111/1365-2664.12319>

16. Bled F, Nichols JD, Altwegg R. Dynamic occupancy models for analyzing species' range dynamics across large geographic scales. Ecology and evolution. 2013; 3: 4896-4909. <https://doi.org/10.1002/ece3.858>

17. Bonnet‐Lebrun AS, Karamanlidis A, de Gabriel Hernando M, Renner I, Gimenez O. Identifying priority conservation areas for a recovering brown bear population in Greece using citizen science data. Animal Conservation. 2019. <https://doi.org/10.1111/acv.12522>

18. Botella C, Joly A, Bonnet P, Monestiez P, Munoz F. Species distribution modeling based on the automated identification of citizen observations. Applications in Plant Sciences. 2018; 6: e1029. <https://doi.org/10.1002/aps3.1029>

19. Boyle WA, Sigel BJ. Ongoing changes in the avifauna of La Selva Biological Station, Costa Rica: twenty-three years of Christmas bird counts. Biological Conservation. 2015; 188: 11-21. <https://doi.org/10.1016/j.biocon.2015.01.004>

20. Bradsworth N, White JG, Isaac B, Cooke R. Species distribution models derived from citizen science data predict the fine scale movements of owls in an urbanizing landscape. Biological conservation. 2017; 213: 27-35. <https://doi.org/10.1016/j.biocon.2017.06.039>

21. Bradter U, Mair L, Jönsson M, Knape J, Singer A, Snäll T. Can opportunistically collected Citizen Science data fill a data gap for habitat suitability models of less common species? Methods in Ecology and Evolution. 2018; 9: 1667-1678. <https://doi.org/10.1111/2041-210x.13012>

22. Breininger DR, Stolen ED, Breininger DJ, Breininger RD. Sampling rare and elusive species: Florida east coast diamondback terrapin population abundance. Ecosphere. 2019; 10: e02824. <https://doi.org/10.1002/ecs2.2824>

23. Bried JT, Siepielski AM. Opportunistic data reveal widespread species turnover in Enallagma damselflies at biogeographical scales. Ecography. 2018; 41: 958-970. <https://doi.org/10.1111/ecog.03419>

24. Brommer J, Alakoski R, Selonen V, Kauhala K. Population dynamics of two beaver species in Finland inferred from citizen‐science census data. Ecosphere. 2017; 8. <https://doi.org/10.1002/ecs2.1947>

25. Broms KM, Johnson DS, Altwegg R, Conquest LL. Spatial occupancy models applied to atlas data show Southern Ground Hornbills strongly depend on protected areas. Ecological Applications. 2014; 24: 363-374. <https://doi.org/10.1890/12-2151.1>

26. Buldrini F, Simoncelli A, Accordi S, Pezzi G, Dallai D. Ten years of citizen science data collection of wetland plants in an urban protected area. Acta Botanica Gallica. 2015; 162: 365-373. <https://doi.org/10.1080/12538078.2015.1080187>

27. Butler CJ, Stanila BD, Iverson JB, Stone PA, Bryson M. Projected changes in climatic suitability for Kinosternon turtles by 2050 and 2070. Ecology and evolution. 2016; 6: 7690-7705. <https://doi.org/10.1002/ece3.2492>

28. Camacho C. Birding trip reports as a data source for monitoring rare species. Animal Conservation. 2016; 19: 430-445. <https://doi.org/10.1111/acv.12258>

29. Campbell H, Engelbrecht I. The Baboon Spider Atlas–using citizen science and the ‘fear factor’ to map baboon spider (Araneae: Theraphosidae) diversity and distributions in Southern Africa. Insect Conservation and Diversity. 2018; 11: 143-151. <https://doi.org/10.1111/icad.12278>

30. Cantú‐Salazar L, Gaston KJ. Species richness and representation in protected areas of the Western hemisphere: discrepancies between checklists and range maps. Divers Distrib. 2013; 19: 782-793. <https://doi.org/10.1111/ddi.12034>

31. Cerrano C, Milanese M, Ponti M. Diving for science‐science for diving: volunteer scuba divers support science and conservation in the Mediterranean Sea. Aquat Conserv. 2017; 27: 303-323. <https://doi.org/10.1002/aqc.2663>

32. de Sá NC, Marchante H, Marchante E, Cabral JA, Honrado JP, Vicente JR. Can citizen science data guide the surveillance of invasive plants? A model-based test with Acacia trees in Portugal. Biological Invasions. 2019; 21: 2127-2141. <https://doi.org/10.1007/s10530-019-01962-6>

33. Champion C, Hobday AJ, Tracey SR, Pecl GT. Rapid shifts in distribution and high‐latitude persistence of oceanographic habitat revealed using citizen science data from a climate change hotspot. Global change biology. 2018; 24: 5440-5453. <https://doi.org/10.1111/gcb.14398>

34. Clare JD, Townsend PA, Anhalt‐Depies C, Locke C, Stenglein JL, Frett S, et al. Making inference with messy (citizen science) data: when are data accurate enough and how can they be improved? Ecological Applications. 2019; 29: e01849. <https://doi.org/10.1002/eap.1849>

35. Colino-Rabanal VJ, Peris SJ. Wildlife roadkills: improving knowledge about ungulate distributions? Hystrix. 2016; 27.

36. Collins SD, Abbott JC, McIntyre NE. Quantifying the degree of bias from using county‐scale data in species distribution modeling: Can increasing sample size or using county‐averaged environmental data reduce distributional overprediction? Ecology and evolution. 2017; 7: 6012-6022. <https://doi.org/10.1002/ece3.3115>

37. Coxen CL, Frey JK, Carleton SA, Collins DP. Species distribution models for a migratory bird based on citizen science and satellite tracking data. Global ecology and conservation. 2017; 11: 298-311. <https://doi.org/10.1016/j.gecco.2017.08.001>

38. Crall AW, Jarnevich CS, Young NE, Panke BJ, Renz M, Stohlgren TJ. Citizen science contributes to our knowledge of invasive plant species distributions. Biol Invasions. 2015; 17: 2415-2427. <https://doi.org/10.1007/s10530-015-0885-4>

39. Crewe TL, Mitchell GW, Larrivée M. Size of the Canadian breeding population of monarch butterflies is driven by factors acting during spring migration and recolonization. Frontiers in Ecology and Evolution. 2019; 7: 308. <https://doi.org/10.3389/fevo.2019.00308>

40. Croft S, Ward AI, Aegerter JN, Smith GC. Modeling current and potential distributions of mammal species using presence‐only data: A case study on British deer. Ecology and Evolution. 2019; 9: 8724-8735. <https://doi.org/10.1002/ece3.5424>

41. Crone EE, Pelton EM, Brown LM, Thomas CC, Schultz CB. Why are monarch butterflies declining in the West? Understanding the importance of multiple correlated drivers. Ecological Applications. 2019: e01975. <https://doi.org/10.1002/bes2.1602>

42. Crum NJ, Fuller AK, Sutherland CS, Cooch EG, Hurst J. Estimating occupancy probability of moose using hunter survey data. The Journal of Wildlife Management. 2017; 81: 521-534. <https://doi.org/10.1002/jwmg.21207>

43. Davis AY, Malas N, Minor ES. Substitutable habitats? The biophysical and anthropogenic drivers of an exotic bird’s distribution. Biological Invasions. 2014; 16: 415-427. <https://doi.org/10.1007/s10530-013-0530-z>

44. De Coster G, De Laet J, Vangestel C, Adriaensen F, Lens L. Citizen science in action—Evidence for long-term, region-wide House Sparrow declines in Flanders, Belgium. Landscape and urban planning. 2015; 134: 139-146. <https://doi.org/10.1016/j.landurbplan.2014.10.020>

45. de Medeiros C, Hernández-Lambraño R, Agudo JS. How Reliable is the Untrained Eye in the Identification of an Invasive Species? The Case of Alien Bee-Hawking Yellow-Legged Hornet in Iberian Peninsula. Contemporary Problems of Ecology. 2018; 11: 666-681. <https://doi.org/10.1134/s1995425518060136>

46. De Rock P, Elwen SH, Roux J, Leeney R, James B, Visser V, et al. Predicting large-scale habitat suitability for cetaceans off Namibia using MinxEnt. Marine Ecology Progress Series. 2019; 619: 149-167. <https://doi.org/10.3354/meps12934>

47. de Sá Dechoum M, Giehl ELH, Sühs RB, Silveira TCL, Ziller SR. Citizen engagement in the management of non-native invasive pines: Does it make a difference? Biological Invasions. 2019: 1-14. <https://doi.org/10.1007/s10530-018-1814-0>

48. De Solan T, Renner I, Cheylan M, Geniez P, Barnagaud JY. Opportunistic records reveal Mediterranean reptiles’ scale‐dependent responses to anthropogenic land use. Ecography. 2018. <https://doi.org/10.1111/ecog.04122>

49. Dennhardt AJ, Duerr AE, Brandes D, Katzner TE. Integrating citizen-science data with movement models to estimate the size of a migratory Golden Eagle population. Biological Conservation. 2015; 184: 68-78. <https://doi.org/10.1016/j.biocon.2015.01.003>

50. Dennis EB, Brereton T, Morgan BJ, Fox R, Shortall CR, Prescott T, et al. Trends and indicators for quantifying moth abundance and occupancy in Scotland. Journal of Insect Conservation. 2019; 23: 369-380. <https://doi.org/10.1007/s10841-019-00135-z>

51. Dennis EB, Morgan BJ, Brereton TM, Roy DB, Fox R. Using citizen science butterfly counts to predict species population trends. Conservation Biology. 2017; 31: 1350-1361. <https://doi.org/10.1111/cobi.12956>

52. Derville S, Torres LG, Iovan C, Garrigue C. Finding the right fit: Comparative cetacean distribution models using multiple data sources and statistical approaches. Diversity and Distributions. 2018; 24: 1657-1673. <https://doi.org/10.1111/ddi.12782>

53. Desaegher J, Nadot S, Machon N, Colas B. How does urbanization affect the reproductive characteristics and ecological affinities of street plant communities? Ecology and evolution. 2019; 9: 9977-9989. <https://doi.org/10.1002/ece3.5539>

54. Deutsch C, Bilenca B, Agostini G. In search of the horned frog (Ceratophrys ornata) in Argentina: complementing field surveys with citizen science. Herpetological Conservation and Biology. 2017; 12: 664-672.

55. Dilts T, Steele M, Engler JD, Pelton EM, Jepsen SJ, McKnight S, et al. Host plants and climate structure habitat associations of the western monarch butterfly. Frontiers in Ecology and Evolution. 2019; 7: 188. <https://doi.org/10.3389/fevo.2019.00188>

56. Dissanayake RB, Stevenson M, Allavena R, Henning J. The value of long-term citizen science data for monitoring koala populations. Scientific reports. 2019; 9: 10037. <https://doi.org/10.1038/s41598-019-46376-5>

57. Dörler D, Kropf M, Laaha G, Zaller JG. Occurrence of the invasive Spanish slug in gardens: can a citizen science approach help deciphering underlying factors? BMC Ecology. 2018; 18: 23. <https://doi.org/10.1186/s12898-018-0179-7>

58. Droz B, Arnoux R, Bohnenstengel T, Laesser J, Spaar R, Ayé R, et al. Moderately urbanized areas as a conservation opportunity for an endangered songbird. Landscape and Urban Planning. 2019; 181: 1-9. <https://doi.org/10.1016/j.landurbplan.2018.09.011>

59. Dunn EH. Dynamics and population consequences of irruption in the Red-breasted Nuthatch (Sitta canadensis). The Auk: Ornithological Advances. 2019; 136: ukz008.

60. Edgar GJ, Ward TJ, Stuart‐Smith RD. Rapid declines across Australian fishery stocks indicate global sustainability targets will not be achieved without an expanded network of ‘no‐fishing’ reserves. Aquatic Conservation: Marine and Freshwater Ecosystems. 2018; 28: 1337-1350. <https://doi.org/10.1002/aqc.2934>

61. Evangelista PH, Mohamed AM, Hussein IA, Saied AH, Mohammed AH, Young NE. Integrating indigenous local knowledge and species distribution modeling to detect wildlife in Somaliland. Ecosphere. 2018; 9. <https://doi.org/10.1002/ecs2.2134>

62. Evans TR, Salvatore D, van de Pol M, Musters C. Adult firefly abundance is linked to weather during the larval stage in the previous year. Ecological Entomology. 2019; 44: 265-273. <https://doi.org/10.1111/een.12702>

63. Fabrizio M, Di Febbraro M, Loy A. Where will it cross next? Optimal management of road collision risk for otters in Italy. Journal of environmental management. 2019; 251: 109609. <https://doi.org/10.1016/j.jenvman.2019.109609>

64. Fink D, Hochachka WM, Zuckerberg B, Winkler DW, Shaby B, Munson MA, et al. Spatiotemporal exploratory models for broad‐scale survey data. Ecological Applications. 2010; 20: 2131-2147. <https://doi.org/10.1890/09-1340.1>

65. Flaherty M, Lawton C. The regional demise of a non-native invasive species: the decline of grey squirrels in Ireland. Biological Invasions. 2019; 21: 2401-2416. <https://doi.org/10.1007/s10530-019-01987-x>

66. Flesch EP, Belt JJ. Comparing citizen science and professional data to evaluate extrapolated mountain goat distribution models. Ecosphere. 2017; 8. <https://doi.org/10.1002/ecs2.1638>

67. Fournier AM, Drake KL, Tozer DC. Using citizen science monitoring data in species distribution models to inform isotopic assignment of migratory connectivity in wetland birds. Journal of avian biology. 2017; 48: 1556-62. <https://doi.org/10.1111/jav.01273>

68. Gange AC, Allen LP, Nussbaumer A, Gange EG, Andrew C, Egli S, et al. Multiscale patterns of rarity in fungi, inferred from fruiting records. Global Ecology and Biogeography. 2019; 28: 1106-1117. <https://doi.org/10.1111/geb.12918>

69. Girado-Beltrán P, Andreu J, Pino J. Exploring changes in the invasion pattern of alien flora in Catalonia (NE of Spain) from large datasets. Biological invasions. 2015; 17: 3015-3028. <https://doi.org/10.1007/s10530-015-0930-3>

70. Girardello M, Chapman A, Dennis R, Kaila L, Borges PA, Santangeli A. Gaps in butterfly inventory data: A global analysis. Biological Conservation. 2019; 236: 289-295. <https://doi.org/10.1016/j.biocon.2019.05.053>

71. Giroux J-F, Patenaude-Monette M, Lagarde F, Mousseau P, Racine F. Changes in spring arrival date and timing of breeding of Ring-billed Gulls in southern Québec over four decades. Avian Conservation and Ecology. 2016; 11. <https://doi.org/10.5751/ace-00821-110101>

72. Goodenough AE. Effects of habitat on breeding success in a declining migrant songbird: the case of Pied Flycatcher Ficedula hypoleuca. Acta ornithologica. 2014; 49: 157-173. <https://doi.org/10.3161/173484714x687046>

73. Goodwin CE, Hodgson DJ, Al‐Fulaij N, Bailey S, Langton S, Mcdonald RA. Voluntary recording scheme reveals ongoing decline in the United Kingdom hazel dormouse Muscardinus avellanarius population. Mammal review. 2017; 47: 183-197. <https://doi.org/10.1111/mam.12091>

74. Gorta SB, Smith JA, Everett JD, Kingsford RT, Cornwell WK, Suthers IM, et al. Pelagic citizen science data reveal declines of seabirds off south-eastern Australia. Biological Conservation. 2019; 235: 226-235. <https://doi.org/10.1016/j.biocon.2019.05.007>

75. Goswami VR, Medhi K, Nichols JD, Oli MK. Mechanistic understanding of human–wildlife conflict through a novel application of dynamic occupancy models. Conservation Biology. 2015; 29: 1100-1110. <https://doi.org/10.1111/cobi.12475>

76. Grüss A, Drexler MD, Chancellor E, Ainsworth CH, Gleason JS, Tirpak JM, et al. Representing species distributions in spatially-explicit ecosystem models from presence-only data. Fisheries Research. 2019; 210: 89-105. <https://doi.org/10.1016/j.fishres.2018.10.011>

77. Hackworth ZJ, Cox JJ, Felch JM, Weegman MD. A Growing Conspiracy: Recolonization of Common Ravens (Corvus corax) in Central and Southern Appalachia, USA. Southeastern Naturalist. 2019; 18: 281-296. <https://doi.org/10.1656/058.018.0208>

78. Hahn NG, Kaufman AJ, Rodriguez-Saona C, Nielsen AL, LaForest J, Hamilton GC. Exploring the spread of brown marmorated stink bug in New Jersey through the use of crowdsourced reports. American Entomologist. 2016; 62: 36-45. <https://doi.org/10.1093/ae/tmw007>

79. Hallworth MT, Sillett TS, Van Wilgenburg SL, Hobson KA, Marra PP. Migratory connectivity of a Neotropical migratory songbird revealed by archival light‐level geolocators. Ecological Applications. 2015; 25: 336-347. <https://doi.org/10.1890/14-0195.1>

80. Hansen BD, Menkhorst P, Moloney P, Loyn RH. Long‐term declines in multiple waterbird species in a tidal embayment, south‐east Australia. Austral Ecology. 2015; 40: 515-527. <https://doi.org/10.1111/aec.12219>

81. Hart AG, Hesselberg T, Nesbit R, Goodenough AE. The spatial distribution and environmental triggers of ant mating flights: using citizen‐science data to reveal national patterns. Ecography. 2018; 41: 877-888. <https://doi.org/10.1111/ecog.03140>

82. Hertzog LR, Besnard A, Jay‐Robert P. Field validation shows bias‐corrected pseudo‐absence selection is the best method for predictive species‐distribution modelling. Diversity and distributions. 2014; 20: 1403-1413. <https://doi.org/10.1111/ddi.12249>

83. Hieb EE, Carmichael RH, Aven A, Nelson-Seely C, Taylor N. Sighting demographics of the West Indian manatee Trichechus manatus in the north-central Gulf of Mexico supported by citizen-sourced data. Endangered Species Research. 2017; 32: 321-332. <https://doi.org/10.3354/esr00817>

84. Higa M, Yamaura Y, Koizumi I, Yabuhara Y, Senzaki M, Ono S. Mapping large‐scale bird distributions using occupancy models and citizen data with spatially biased sampling effort. Diversity and Distributions. 2015; 21: 46-54. <https://doi.org/10.1111/ddi.12255>

85. Hill JM, Lloyd JD. A fine‐scale US population estimate of a montane spruce–fir bird species of conservation concern. Ecosphere. 2017; 8. <https://doi.org/10.1002/ecs2.1921>

86. Horns JJ, Adler FR, Şekercioğlu ÇH. Using opportunistic citizen science data to estimate avian population trends. Biological conservation. 2018; 221: 151-159. <https://doi.org/10.1016/j.biocon.2018.02.027>

87. Horton KG, Van Doren BM, La Sorte FA, Cohen EB, Clipp HL, Buler JJ, et al. Holding steady: Little change in intensity or timing of bird migration over the Gulf of Mexico. Global change biology. 2019; 25: 1106-1118. <https://doi.org/10.1111/gcb.14540>

88. Hosseini M, Farashi A, Khani A, Farhadinia MS. Landscape connectivity for mammalian megafauna along the Iran-Turkmenistan-Afghanistan borderland. Journal for Nature Conservation. 2019; 52: 125735. <https://doi.org/10.1016/j.jnc.2019.125735>

89. Howes C, Symes CT, Byholm P. Evidence of large‐scale range shift in the distribution of a Palaearctic migrant in Africa. Diversity and Distributions. 2019. <https://doi.org/10.1111/ddi.12922>

90. Hugo S, Altwegg R. The second Southern African Bird Atlas Project: causes and consequences of geographical sampling bias. Ecology and evolution. 2017; 7: 6839-6849. <https://doi.org/10.1002/ece3.3228>

91. Humphreys JM, Murrow JL, Sullivan JD, Prosser DJ. Seasonal occurrence and abundance of dabbling ducks across the continental United States: Joint spatio‐temporal modelling for the Genus Anas. Diversity and Distributions. 2019. <https://doi.org/10.1111/ddi.12960>

92. Ivanova IM, Symes CT. Invasion of Psittacula krameri in Gauteng, South Africa: are other birds impacted? Biodiversity and Conservation. 2019: 1-24. <https://doi.org/10.1007/s10531-019-01841-5>

93. Jackson MM, Gergel SE, Martin K. Citizen science and field survey observations provide comparable results for mapping Vancouver Island White-tailed Ptarmigan (Lagopus leucura saxatilis) distributions. Biological Conservation. 2015; 181: 162-172. <https://doi.org/10.1016/j.biocon.2014.11.010>

94. Jiguet F, Devictor V, Julliard R, Couvet D. French citizens monitoring ordinary birds provide tools for conservation and ecological sciences. Acta Oecologica. 2012; 44: 58-66. <https://doi.org/10.1016/j.actao.2011.05.003>

95. Jiménez‐Valverde A, Peña‐Aguilera P, Barve V, Burguillo‐Madrid L. Photo‐sharing platforms key for characterising niche and distribution in poorly studied taxa. Insect Conservation and Diversity. 2019; 12: 389-403. <https://doi.org/10.1111/icad.12351>

96. Johnston A, Fink D, Hochachka WM, Kelling S. Estimates of observer expertise improve species distributions from citizen science data. Methods in Ecology and Evolution. 2018; 9: 88-97. <https://doi.org/10.1111/2041-210x.12838>

97. Jones KE, Russ JA, Bashta AT, Bilhari Z, Catto C, Csősz I, et al. Indicator bats program: a system for the global acoustic monitoring of bats. Biodiversity monitoring and conservation: bridging the gap between global commitment and local action. 2013: 211-247. <https://doi.org/10.1002/9781118490747.ch10>

98. Kamp J, Oppel S, Heldbjerg H, Nyegaard T, Donald PF. Unstructured citizen science data fail to detect long‐term population declines of common birds in Denmark. Diversity and Distributions. 2016; 22: 1024-1035. <https://doi.org/10.1111/ddi.12463>

99. Kasahara S, Koyama K. Population trends of common wintering waterfowl in Japan: participatory monitoring data from 1996 to 2009. Ornithological science. 2010; 9: 23-36. <https://doi.org/10.2326/osj.9.23>

100. Kéry M, Gardner B, Monnerat C. Predicting species distributions from checklist data using site‐occupancy models. Journal of Biogeography. 2010; 37: 1851-1862. <https://doi.org/10.1111/j.1365-2699.2010.02345.x>

101. Kery M, Royle JA, Schmid H, Schaub M, Volet B, Haefliger G, et al. Site‐occupancy distribution modeling to correct population‐trend estimates derived from opportunistic observations. Conservation Biology. 2010; 24: 1388-1397. <https://doi.org/10.1111/j.1523-1739.2010.01479.x>

102. Koparde P, Mehta P, Mukherjee S, Robin V. Quaternary climatic fluctuations and resulting climatically suitable areas for Eurasian owlets. Ecology and evolution. 2019; 9: 4864-4874. <https://doi.org/10.1002/ece3.5086>

103. Kreling SE, Gaynor KM, Coon CA. Roadkill distribution at the wildland‐urban interface. The Journal of Wildlife Management. 2019; 83: 1427-1436. <https://doi.org/10.1002/jwmg.21692>

104. Krolikowska N, Krupinski D, Kuczynski L. Combining data from multiple sources to design a raptor census-the first national survey of the Montagu’s Harrier Circus pygargus in Poland. Bird Conservation International. 2018; 28: 350-362. <https://doi.org/10.1017/s0959270917000235>

105. La Sorte FA, Fink D, Blancher PJ, Rodewald AD, Ruiz‐Gutierrez V, Rosenberg KV, et al. Global change and the distributional dynamics of migratory bird populations wintering in Central America. Global change biology. 2017; 23: 5284-5296. <https://doi.org/10.1111/gcb.13794>

106. La Sorte FA, Fink D, Johnston A. Time of emergence of novel climates for North American migratory bird populations. Ecography. 2019. <https://doi.org/10.1111/ecog.04408>

107. La Sorte FA, Fink D, Hochachka WM, Aycrigg JL, Rosenberg KV, Rodewald AD, et al. Documenting stewardship responsibilities across the annual cycle for birds on US public lands. Ecological Applications. 2015; 25: 39-51. <https://doi.org/10.1890/14-0702.1>

108. La Sorte FA, Hochachka WM, Farnsworth A, Dhondt AA, Sheldon D. The implications of mid‐latitude climate extremes for North American migratory bird populations. Ecosphere. 2016; 7. <https://doi.org/10.1002/ecs2.1261>

109. Laughlin AJ, Sheldon DR, Winkler DW, Taylor CM. Quantifying non‐breeding season occupancy patterns and the timing and drivers of autumn migration for a migratory songbird using Doppler radar. Ecography. 2016; 39: 1017-1024. <https://doi.org/10.1111/ecog.01988>

110. Laughlin AJ, Taylor CM, Bradley DW, Leclair D, Clark RC, Dawson RD, et al. Integrating information from geolocators, weather radar, and citizen science to uncover a key stopover area of an aerial insectivore. The Auk. 2013; 130: 230-239. <https://doi.org/10.1525/auk.2013.12229>

111. Lees AC, Martin RW. Exposing hidden endemism in a Neotropical forest raptor using citizen science. Ibis. 2015; 157: 103-114. <https://doi.org/10.1111/ibi.12207>

112. Lin Y-P, Deng D, Lin W-C, Lemmens R, Crossman ND, Henle K, et al. Uncertainty analysis of crowd-sourced and professionally collected field data used in species distribution models of Taiwanese moths. Biological conservation. 2015; 181: 102-110. <https://doi.org/10.1016/j.biocon.2014.11.012>

113. Long AM, Pierce BL, Anderson AD, Skow KL, Smith A, Lopez RR. Integrating citizen science and remotely sensed data to help inform time-sensitive policy decisions for species of conservation concern. Biological Conservation. 2019; 237: 463- 469. <https://doi.org/10.1016/j.biocon.2019.07.025>

114. Looney C, Strange JP, Freeman M, Jennings D. The expanding Pacific Northwest range of Bombus impatiens Cresson and its establishment in Washington State. Biological Invasions. 2019; 21: 1879-1885. <https://doi.org/10.1007/s10530-019-01970-6>

115. Louvrier J, Molinari‐Jobin A, Kéry M, Chambert T, Miller D, Zimmermann F, et al. Use of ambiguous detections to improve estimates from species distribution models. Conservation Biology. 2019; 33: 185-195. <https://doi.org/10.1111/cobi.13191>

116. Nimis PL, Pittao E, Altobelli A, De Pascalis F, Laganis J, Martellos S. Mapping invasive plants with citizen science. A case study from Trieste (NE Italy). Plant Biosystems. 2018: 1-10. <https://doi.org/10.1080/11263504.2018.1536085>

117. Luizza M, Wakie T, Evangelista P, Jarnevich C. Integrating local pastoral knowledge, participatory mapping, and species distribution modeling for risk assessment of invasive rubber vine (Cryptostegia grandiflora) in Ethiopia’s Afar region. Ecology and Society. 2016; 21. <https://doi.org/10.5751/es-07988-210122>

118. Lyon JP, Bird TJ, Kearns J, Nicol S, Tonkin Z, Todd CR, et al. Increased population size of fish in a lowland river following restoration of structural habitat. Ecological Applications. 2019: e01882. <https://doi.org/10.1002/eap.1882>

119. MacPhail VJ, Richardson LL, Colla SR. Incorporating citizen science, museum specimens, and field work into the assessment of extinction risk of the American Bumble bee (Bombus pensylvanicus De Geer 1773) in Canada. Journal of Insect Conservation. 2019; 23: 597-611. <https://doi.org/10.1007/s10841-019-00152-y>

120. Mair L, Harrison PJ, Jönsson M, Löbel S, Nordén J, Siitonen J, et al. Evaluating citizen science data for forecasting species responses to national forest management. Ecology and evolution. 2017; 7: 368-378. <https://doi.org/10.1002/ece3.2601>

121. Marsh DM, Cosentino BJ. Causes and consequences of non-random drop-outs for citizen science projects: lessons from the North American amphibian monitoring program. Freshwater Science. 2019; 38: 292-302. <https://doi.org/10.1086/701672>

122. Marsh DM, Cosentino BJ, Jones KS, Apodaca JJ, Beard KH, Bell JM, et al. Effects of roads and land use on frog distributions across spatial scales and regions in the Eastern and Central United States. Diversity and Distributions. 2017; 23: 158-170. <https://doi.org/10.1111/ddi.12516>

123. Martín B, Torralvo CA, Elias G, Tomás J, Onrubia A, Ferrer M. Are Western European ospreys (Pandion haliaetus) shortening their migration distances? Evidence from trends of the wintering population in the Iberian Peninsula. European Journal of Wildlife Research. 2019; 65: 72. <https://doi.org/10.1007/s10344-019-1311-5>

124. Martin G, Devictor V, Motard E, Machon N, Porcher E. Short-term climate-induced change in French plant communities. Biology letters. 2019; 15: 20190280. <https://doi.org/10.1098/rsbl.2019.0280>

125. Marx M, Quillfeldt P. Species distribution models of European Turtle Doves in Germany are more reliable with presence only rather than presence absence data. Scientific reports. 2018; 8: 16898. <https://doi.org/10.1038/s41598-018-35318-2>

126. Mason SC, Hill JK, Thomas CD, Powney GD, Fox R, Brereton T, et al. Population variability in species can be deduced from opportunistic citizen science records: a case study using British butterflies. Insect conservation and diversity. 2018; 11: 131-142. <https://doi.org/10.1111/icad.12242>

127. Massimino D, Harris SJ, Gillings S. Evaluating spatiotemporal trends in terrestrial mammal abundance using data collected during bird surveys. Biological conservation. 2018; 226: 153-167. <https://doi.org/10.1016/j.biocon.2018.07.026>

128. McCoshum SM, Andreoli SL, Stenoien CM, Oberhauser KS, Baum KA. Species distribution models for natural enemies of monarch butterfly (Danaus plexippus) larvae and pupae: distribution patterns and implications for conservation. Journal of insect conservation. 2016; 20: 223-237. <https://doi.org/10.1007/s10841-016-9856-z>

129. McDuffie LA, Hagelin JC, Snively ML, Pendleton GW, Taylor AR. Citizen Science Observations Reveal Long-Term Population Trends of Common and Pacific Loon in Urbanized Alaska. Journal of Fish and Wildlife Management. 2019; 10: 148-162. <https://doi.org/10.3996/082018-naf-002>

130. Meehan TD, Michel NL, Rue H. Spatial modeling of Audubon Christmas Bird Counts reveals fine‐scale patterns and drivers of relative abundance trends. Ecosphere. 2019; 10: e02707. <https://doi.org/10.1002/ecs2.2707>

131. Melero Y, Stefanescu C, Pino J. General declines in Mediterranean butterflies over the last two decades are modulated by species traits. Biological Conservation. 2016; 201: 336-342. <https://doi.org/10.1016/j.biocon.2016.07.029>

132. Miller R, Paprocki N, Stuber M, Moulton C, Carlisle J. Short-eared Owl (Asio flammeus) surveys in the North American Intermountain West: utilizing citizen scientists to conduct monitoring across a broad geographic scale. Avian Conservation and Ecology. 2016; 11. <https://doi.org/10.5751/ace-00819-110103>

133. Mims MC, Olson DH, Pilliod DS, Dunham JB. Functional and geographic components of risk for climate sensitive vertebrates in the Pacific Northwest, USA. Biological Conservation. 2018; 228: 183-194. <https://doi.org/10.1016/j.biocon.2018.10.012>

134. Mingozzi T, Storino P, Venuto G, Alessandria G, Arcamone E, Urso S, et al. Autumn migration of Common Cranes Grus grus through the Italian Peninsula: new vs. historical flyways and their meteorological correlates. Acta Ornithologica. 2013; 48: 165-177. <https://doi.org/10.3161/000164513x678810>

135. Mohanty NP, Measey J. Reconstructing biological invasions using public surveys: a new approach to retrospectively assess spatio-temporal changes in invasive spread. Biological Invasions. 2018: 1-14. <https://doi.org/10.1007/s10530-018-1839-4>

136. Mohanty NP, Sachin A, Selvaraj G, Vasudevan K. Using public surveys to reliably and rapidly estimate the distributions of multiple invasive species on the Andaman archipelago. Biotropica. 2018; 50: 197-201. <https://doi.org/10.1111/btp.12534>

137. Molinari‐Jobin A, Kéry M, Marboutin E, Marucco F, Zimmermann F, Molinari P, et al. Mapping range dynamics from opportunistic data: spatiotemporal modelling of the lynx distribution in the Alps over 21 years. Animal conservation. 2018; 21: 168-180. <https://doi.org/10.1111/acv.12369>

138. Monsarrat S, Boshoff AF, Kerley GI. Accessibility maps as a tool to predict sampling bias in historical biodiversity occurrence records. Ecography. 2018; 42: 125-136. <https://doi.org/10.1111/ecog.03944>

139. Morii Y, Nakano T. Citizen science reveals the present range and a potential native predator of the invasive slug Limax maximus Linnæus, 1758 in Hokkaido, Japan. Bioinvasions Rec. 2017; 6: 181-186. <https://doi.org/10.3391/bir.2017.6.3.01>

140. Mosnier A, Gosselin J-F, Lawson J, Plourde S, Lesage V. Predicting seasonal occurrence of leatherback turtles (Dermochelys coriacea) in eastern Canadian waters from turtle and ocean sunfish (Mola mola) sighting data and habitat characteristics. Canadian Journal of Zoology. 2018; 97: 464-478. <https://doi.org/10.1139/cjz-2018-0167>

141. Musilová Z, Musil P, Zouhar J, Adam M. Changes in habitat suitability influence non‐breeding distribution of waterbirds in central Europe. Ibis. 2018; 160: 582-596. <https://doi.org/10.1111/ibi.12559>

142. Musilová Z, Musil P, Zouhar J, Adam M, Bejček V. Importance of Natura 2000 sites for wintering waterbirds: Low preference, species' distribution changes and carrying capacity of Natura 2000 could fail to protect the species. Biological Conservation. 2018; 228: 79-88. <https://doi.org/10.1016/j.biocon.2018.10.004>

143. Nagy C, Bardwell K, Rockwell RF, Christie R, Weckel M. Validation of a citizen science-based model of site occupancy for eastern screech owls with systematic data in suburban New York and Connecticut. Northeastern Naturalist. 2012; 19: 143-159. <https://doi.org/10.1656/045.019.s611>

144. Newson SE, Moran NJ, Musgrove AJ, Pearce‐Higgins JW, Gillings S, Atkinson PW, et al. Long‐term changes in the migration phenology of UK breeding birds detected by large‐scale citizen science recording schemes. Ibis. 2016; 158: 481-495. <https://doi.org/10.1111/ibi.12367>

145. Pace DS, Giacomini G, Campana I, Paraboschi M, Pellegrino G, Silvestri M, et al. An integrated approach for cetacean knowledge and conservation in the central Mediterranean Sea using research and social media data sources. Aquatic Conservation: Marine and Freshwater Ecosystems. 2019; 29: 1302-1323. <https://doi.org/10.1002/aqc.3117>

146. Pacifici K, Reich BJ, Miller DA, Pease BS. Resolving misaligned spatial data with integrated species distribution models. Ecology. 2019: e02709. <https://doi.org/10.1002/ecy.2709>

147. Pagel J, Anderson BJ, O'Hara RB, Cramer W, Fox R, Jeltsch F, et al. Quantifying range‐wide variation in population trends from local abundance surveys and widespread opportunistic occurrence records. Methods in Ecology and Evolution. 2014; 5: 751-760. <https://doi.org/10.1111/2041-210X.12221>

148. Paukkunen J, Pöyry J, Kuussaari M. Species traits explain long‐term population trends of Finnish cuckoo wasps (Hymenoptera: Chrysididae). Insect Conservation and Diversity. 2018; 11: 58-71. <https://doi.org/10.1111/icad.12241>

149. Peach MA, Cohen JB, Frair JL. Single‐visit dynamic occupancy models: an approach to account for imperfect detection with Atlas data. Journal of applied ecology. 2017; 54: 2033-2042. <https://doi.org/10.1111/1365-2664.12925>

150. Peña-Aguilera P, Burguillo-Madrid L, Barve V, Aragón P, Jiménez-Valverde A. Niche segregation in Iberian Argiope species. The Journal of Arachnology. 2019; 47: 37-44. <https://doi.org/10.1636/0161-8202-47.1.37>

151. Péron G, Altwegg R. Low bird diversity in the Fynbos plant diversity hotspot: Quaternary legacies in the current distributions of passerine birds. Ecography. 2015; 38: 992-997. <https://doi.org/10.1111/ecog.01176>

152. Péron G, Altwegg R. Twenty‐five years of change in southern African passerine diversity: nonclimatic factors of change. Global change biology. 2015; 21: 3347-3355. <https://doi.org/10.1111/gcb.12909>

153. Pescott OL, Simkin JM, August TA, Randle Z, Dore AJ, Botham MS. Air pollution and its effects on lichens, bryophytes, and lichen-feeding Lepidoptera: review and evidence from biological records. Biological Journal of the Linnean Society. 2015; 115: 611-635. <https://doi.org/10.1111/bij.12541>

154. Phillips ND, Reid N, Thys T, Harrod C, Payne NL, Morgan CA, et al. Applying species distribution modelling to a data poor, pelagic fish complex: The ocean sunfishes. Journal of biogeography. 2017; 44: 2176-2187. <https://doi.org/10.1111/jbi.13033>

155. Pillay R, Miller DA, Hines JE, Joshi AA, Madhusudan M. Accounting for false positives improves estimates of occupancy from key informant interviews. Diversity and Distributions. 2014; 20: 223-235. <https://doi.org/10.1111/ddi.12151>

156. Plummer KE, Siriwardena GM, Conway GJ, Risely K, Toms MP. Is supplementary feeding in gardens a driver of evolutionary change in a migratory bird species? Global Change Biology. 2015; 21: 4353-4563. <https://doi.org/10.1111/gcb.13070>

157. Prodon R, Geniez P, Cheylan M, Devers F, Chuine I, Besnard A. A reversal of the shift towards earlier spring phenology in several Mediterranean reptiles and amphibians during the 1998–2013 warming slowdown. Global change biology. 2017; 23: 5481-5491. <https://doi.org/10.1111/gcb.13812>

158. Puan CL, Yeong KL, Ong KW, Fauzi MIA, Yahya MS, Khoo SS. Influence of landscape matrix on urban bird abundance: evidence from Malaysian citizen science data. Journal of Asia-Pacific Biodiversity. 2019. <https://doi.org/10.1016/j.japb.2019.03.008>

159. Ralston J, King DI, DeLuca WV, Niemi GJ, Glennon MJ, Scarl JC, et al. Analysis of combined data sets yields trend estimates for vulnerable spruce-fir birds in northern United States. Biological Conservation. 2015; 187: 270-278. <https://doi.org/10.1016/j.biocon.2015.04.029>

160. Rapacciuolo G, Ball-Damerow J, Zeilinger A, Resh V. Detecting long-term occupancy changes in Californian odonates from natural history and citizen science records. Biodiversity and Conservation. 2017; 26: 2933-2949. <https://doi.org/10.1007/s10531-017-1399-4>

161. Reed GC, Litvaitis JA, Ellingwood M, Tate P, Broman DJ, Sirén AP, et al. Describing habitat suitability of bobcats (Lynx rufus) using several sources of information obtained at multiple spatial scales. Mammalian Biology. 2017; 82: 17-26. <https://doi.org/10.1016/j.mambio.2016.10.002>

162. Ribeiro I, Proença V, Serra P, Palma J, Domingo-Marimon C, Pons X, et al. Remotely sensed indicators and open-access biodiversity data to assess bird diversity patterns in Mediterranean rural landscapes. Scientific reports. 2019; 9: 6826. <https://doi.org/10.1101/408187>

163. Richardson SJ, Clayton R, Rance BD, Broadbent H, McGlone MS, Wilmshurst JM. Small wetlands are critical for safeguarding rare and threatened plant species. Applied Vegetation Science. 2015; 18: 230-241. <https://doi.org/10.1111/avsc.12144>

164. Robinson OJ, Ruiz‐Gutierrez V, Fink D. Correcting for bias in distribution modelling for rare species using citizen science data. Diversity and Distributions. 2018; 24: 460-472. <https://doi.org/10.1111/ddi.12698>

165. Robinson RA, Morrison CA, Baillie SR. Integrating demographic data: towards a framework for monitoring wildlife populations at large spatial scales. Methods in Ecology and Evolution. 2014; 5: 1361-1372. <https://doi.org/10.1111/2041-210x.12204>

166. Rodewald AD, Strimas-Mackey M, Schuster R, Arcese P. Beyond canaries in coal mines: Co-occurrence of Andean mining concessions and migratory birds. Perspectives in Ecology and Conservation. 2019; 17: 151-156. <https://doi.org/10.1016/j.pecon.2019.08.002>

167. Rodhouse TJ, Ormsbee PC, Irvine KM, Vierling LA, Szewczak JM, Vierling KT. Establishing conservation baselines with dynamic distribution models for bat populations facing imminent decline. Diversity and Distributions. 2015; 21: 1401-1413. <https://doi.org/10.1111/ddi.12372>

168. Ruete A, Pärt T, Berg Å, Knape J. Exploiting opportunistic observations to estimate changes in seasonal site use: An example with wetland birds. Ecology and evolution. 2017; 7: 5632-5644. <https://doi.org/10.7287/peerj.preprints.2612>

169. Ruiz‐Gutierrez V, Hooten MB, Grant EHC. Uncertainty in biological monitoring: a framework for data collection and analysis to account for multiple sources of sampling bias. Methods in Ecology and Evolution. 2016; 7: 900-909. <https://doi.org/10.1111/2041-210x.12542>

170. Rutten A, Casaer J, Swinnen KR, Herremans M, Leirs H. Future distribution of wild boar in a highly anthropogenic landscape: Models combining hunting bag and citizen science data. Ecological Modelling. 2019; 411: 108804. <https://doi.org/10.1016/j.ecolmodel.2019.108804>

171. Santika T, McAlpine CA, Lunney D, Wilson KA, Rhodes JR. Modelling species distributional shifts across broad spatial extents by linking dynamic occupancy models with public‐based surveys. Diversity and Distributions. 2014; 20: 786-796. <https://doi.org/10.1111/ddi.12189>

172. Sardà-Palomera F, Brotons L, Villero D, Sierdsema H, Newson SE, Jiguet F. Mapping from heterogeneous biodiversity monitoring data sources. Biodiversity and conservation. 2012; 21: 2927-2948. <https://doi.org/10.1007/s10531-012-0347-6>

173. Sequeira AM, Roetman PE, Daniels CB, Baker AK, Bradshaw CJ. Distribution models for koalas in South Australia using citizen science‐collected data. Ecology and Evolution. 2014; 4: 2103-2114. <https://doi.org/10.1002/ece3.1094>

174. Sillero N, Campos J, Bonardi A, Corti C, Creemers R, Crochet P-A, et al. Updated distribution and biogeography of amphibians and reptiles of Europe. Amphibia-Reptilia. 2014; 35: 1-31.

175. Smale DA, Epstein G, Parry M, Attrill MJ. Spatiotemporal variability in the structure of seagrass meadows and associated macrofaunal assemblages in southwest England (UK): Using citizen science to benchmark ecological pattern. Ecology and evolution. 2019; 9: 3958-3972. <https://doi.org/10.1002/ece3.5025>

176. Snäll T, Kindvall O, Nilsson J, Pärt T. Evaluating citizen-based presence data for bird monitoring. Biological conservation. 2011; 144: 804-810. <https://doi.org/10.1016/j.biocon.2010.11.010>

177. Soroye P, Ahmed N, Kerr JT. Opportunistic citizen science data transform understanding of species distributions, phenology, and diversity gradients for global change research. Global change biology. 2018; 24: 5281-5291. <https://doi.org/10.1111/gcb.14358>

178. Soykan CU, Sauer J, Schuetz JG, LeBaron GS, Dale K, Langham GM. Population trends for North American winter birds based on hierarchical models. Ecosphere. 2016; 7: e01351. <https://doi.org/10.1002/ecs2.1351>

179. Stefanescu C, Páramo F, Åkesson S, Alarcón M, Ávila A, Brereton T, et al. Multi‐generational long‐distance migration of insects: studying the painted lady butterfly in the Western Palaearctic. Ecography. 2013; 36: 474-486. <https://doi.org/10.1111/j.1600-0587.2012.07738.x>

180. Sumner S, Bevan P, Hart AG, Isaac NJ. Mapping species distributions in 2 weeks using citizen science. Insect Conservation and Diversity. 2019. <https://doi.org/10.1111/icad.12345>

181. Supp S, La Sorte FA, Cormier TA, Lim MC, Powers DR, Wethington SM, et al. Citizen‐science data provides new insight into annual and seasonal variation in migration patterns. Ecosphere. 2015; 6: 1-19. <https://doi.org/10.1890/es14-00290.1>

182. Sweet LC, Green T, Heintz JG, Frakes N, Graver N, Rangitsch JS, et al. Congruence between future distribution models and empirical data for an iconic species at Joshua Tree National Park. Ecosphere. 2019; 10: e02763. <https://doi.org/10.1002/ecs2.2763>

183. Szabo JK, Vesk PA, Baxter PW, Possingham HP. Regional avian species declines estimated from volunteer‐collected long‐term data using List Length Analysis. Ecological Applications. 2010; 20: 2157-2169. <https://doi.org/10.1890/09-0877.1>

184. Termaat T, van Strien AJ, van Grunsven RH, De Knijf G, Bjelke U, Burbach K, et al. Distribution trends of European dragonflies under climate change. Diversity and Distributions. 2019; 25: 936-950. <https://doi.org/10.1111/ddi.12913>

185. Thorson JT, Scheuerell MD, Semmens BX, Pattengill-Semmens CV. Demographic modeling of citizen science data informs habitat preferences and population dynamics of recovering fishes. Ecology. 2014; 95: 3251-3258. <https://doi.org/10.1890/13-2223.1>

186. Tiago P, Pereira HM, Capinha C. Using citizen science data to estimate climatic niches and species distributions. Basic and Applied Ecology. 2017; 20: 75-85. <https://doi.org/10.1016/j.baae.2017.04.001>

187. Titeux N, Maes D, Van Daele T, Onkelinx T, Heikkinen RK, Romo H, et al. The need for large‐scale distribution data to estimate regional changes in species richness under future climate change. Diversity and Distributions. 2017; 23: 1393-1407. <https://doi.org/10.1111/ddi.12634>

188. Todd BD, Rose JP, Price SJ, Dorcas ME. Using citizen science data to identify the sensitivity of species to human land use. Conservation biology. 2016; 30: 1266-1276. <https://doi.org/10.1111/cobi.12686>

189. Tonachella N, Nastasi A, Kaufman G, Maldini D, Rankin RW. Predicting trends in humpback whale (Megaptera novaeangliae) abundance using citizen science. Pacific Conservation Biology. 2012; 18: 297-309. <https://doi.org/10.1071/pc120297>

190. Tulloch A, Mustin K, Possingham HP, Szabo JK, Wilson KA. To boldly go where no volunteer has gone before: predicting volunteer activity to prioritize surveys at the landscape scale. Diversity and Distributions. 2013; 19: 465-480. <https://doi.org/10.1111/j.1472-4642.2012.00947.x>

191. Tye CA, McCleery RA, Fletcher Jr RJ, Greene DU, Butryn RS. Evaluating citizen vs. professional data for modelling distributions of a rare squirrel. Journal of applied ecology. 2017; 54: 628-637. <https://doi.org/10.1111/1365-2664.12682>

192. van Strien A, Termaat T, Kalkman V, Prins M, De Knijf G, Gourmand A-L, et al. Occupancy modelling as a new approach to assess supranational trends using opportunistic data: a pilot study for the damselfly Calopteryx splendens. Biodiversity and conservation. 2013; 22: 673-686. <https://doi.org/10.1007/s10531-013-0436-1>

193. van Strien AJ, van Swaay CAM, Termaat T. Opportunistic citizen science data of animal species produce reliable estimates of distribution trends if analysed with occupancy models. Journal of Applied Ecology. 2013; 50: 1450-1458. <https://doi.org/10.1111/1365-2664.12158>

194. van Strien AJ, van Swaay CA, van Strien-van Liempt WT, Poot MJ, WallisDeVries MF. Over a century of data reveal more than 80% decline in butterflies in the Netherlands. Biological Conservation. 2019; 234: 116-122. <https://doi.org/10.1016/j.biocon.2019.03.023>

195. Vantieghem P, Maes D, Kaiser A, Merckx T. Quality of citizen science data and its consequences for the conservation of skipper butterflies (Hesperiidae) in Flanders (northern Belgium). Journal of Insect Conservation. 2017; 21: 451-463. <https://doi.org/10.1007/s10841-016-9924-4>

196. Veran S, Piry S, Ternois V, Meynard CN, Facon B, Estoup A. Modeling spatial expansion of invasive alien species: relative contributions of environmental and anthropogenic factors to the spreading of the harlequin ladybird in France. Ecography. 2016; 39: 665-675. <https://doi.org/10.1111/ecog.01389>

197. Villeneuve AR, Thornhill I, Eales J. Upstream migration and altitudinal distribution patterns of Nereina punctulata (Gastropoda: Neritidae) in Dominica, West Indies. Aquatic Ecology. 2019; 53: 205-215. <https://doi.org/10.1007/s10452-019-09683-7>

198. White ER, Myers MC, Flemming JM, Baum JK. Shifting elasmobranch community assemblage at Cocos Island—an isolated marine protected area. Conservation Biology. 2015; 29: 1186-1197. <https://doi.org/10.1111/cobi.12478>

199. Widenfalk LA, Ahrné K, Berggren Å. Using citizen‐reported data to predict distributions of two non‐native insect species in Sweden. Ecosphere. 2014; 5: 1-16. <https://doi.org/10.1890/es14-00212.1>

200. Williams MR, Yates CJ, Stock WD, Barrett GW, Finn HC. Citizen science monitoring reveals a significant, ongoing decline of the Endangered Carnaby's black-cockatoo Calyptorhynchus latirostris. Oryx. 2016; 50: 626-635. <https://doi.org/10.1017/s0030605315000320>

201. Wilson JF, Baker D, Cook M, Davis G, Freestone R, Gardner D, et al. Climate association with fluctuation in annual abundance of fifty widely distributed moths in England and Wales: a citizen-science study. Journal of insect conservation. 2015; 19: 935-946. <https://doi.org/10.1007/s10841-015-9811-4>

202. Wine S, Gagné SA, Meentemeyer RK. Understanding human–coyote encounters in urban ecosystems using citizen science data: what do socioeconomics tell us? Environmental management. 2015; 55: 159-170. <https://doi.org/10.1007/s00267-014-0373-0>

203. Yue S, Bonebrake TC, Gibson L. Informing snake roadkill mitigation strategies in Taiwan using citizen science. The Journal of Wildlife Management. 2019; 83: 80-88. <https://doi.org/10.1002/jwmg.21580>

204. Zapponi L, Cini A, Bardiani M, Hardersen S, Maura M, Maurizi E, et al. Citizen science data as an efficient tool for mapping protected saproxylic beetles. Biological Conservation. 2017; 208: 139-145. <https://doi.org/10.1016/j.biocon.2016.04.035>

205. Zeng Q, Wei Q, Lei G. Contribution of citizen science towards cryptic species census: “many eyes” define wintering range of the Scaly-sided Merganser in mainland China. Avian Research. 2018; 9: 6. <https://doi.org/10.1186/s40657-018-0098-5>

206. Zhang X, Vincent AC. Integrating multiple datasets with species distribution models to inform conservation of the poorly-recorded Chinese seahorses. Biological conservation. 2017; 211: 161-171. <https://doi.org/10.1016/j.biocon.2017.05.020>

207. Zub K, Kozieł M, Siłuch M, Bednarczyk P, Zalewski A. The NATURA 2000 database as a tool in the analysis of habitat selection at large scales: factors affecting the occurrence of pine and stone martens in Southern Europe. European journal of wildlife research. 2018; 64: 10. <https://doi.org/10.1007/s10344-018-1168-z>

208. Bird TJ, Bates AE, Lefcheck JS, Hill NA, Thomson RJ, Edgar GJ, et al. Statistical solutions for error and bias in global citizen science datasets. Biological Conservation. 2014; 173: 144-154. <https://doi.org/10.1016/j.biocon.2013.07.037>.
